# Late Holocene dietary and cultural variability on the Xingu River, Amazon Basin: A stable isotopic approach

**DOI:** 10.1371/journal.pone.0271545

**Published:** 2022-08-03

**Authors:** Letícia Morgana Müller, Renato Kipnis, Mariane Pereira Ferreira, Sara Marzo, Bianca Fiedler, Mary Lucas, Jana Ilgner, Hilton P. Silva, Patrick Roberts

**Affiliations:** 1 Department of Archaeology, Max Planck Institute for Geoanthropology (formerly the Science of Human History), Jena, Germany; 2 Graduate Program in Anthropology, Federal University of Pará, Belém, Pará, Brazil; 3 Scientia Consultoria Científica, São Paulo, São Paulo—SP, Brazil; 4 Graduate Program in Archeology, Museum of Archaeology and Etnology, University of São Paulo, São Paulo, Brazil; 5 The Roslin Institute & Royal (Dick) School of Veterinary Studies, University of Edinburgh, Midlothian, Edinburgh, United Kingdom; 6 isoTROPIC Independent Research Group, Max Planck Institute for Geoanthropology (formerly the Science of Human History), Jena, Germany; 7 School of Social Science, The University of Queensland, Brisbane, QLD, Australia; Ghazi University, PAKISTAN

## Abstract

Although once considered a ‘counterfeit paradise’, the Amazon Basin is now a region of increasing interest in discussions of pre-colonial tropical land-use and social complexity. Archaeobotany, archaeozoology, remote sensing and palaeoecology have revealed that, by the Late Holocene, populations in different parts of the Amazon Basin were using various domesticated plants, modifying soils, building earthworks, and even forming ‘Garden Cities’ along the Amazon River and its tributaries. However, there remains a relatively limited understanding as to how diets, environmental management, and social structures varied across this vast area. Here, we apply stable isotope analysis to human remains (n = 4 for collagen, n = 17 for tooth enamel), and associated fauna (n = 61 for collagen, n = 28 for tooth enamel), to directly determine the diets of populations living in the Volta Grande do Rio Xingu, an important region of pre-Columbian cultural interactions, between 390 cal. years BC and 1,675 cal. years AD. Our results highlight an ongoing dietary focus on C_3_ plants and wild terrestrial fauna and aquatic resources across sites and time periods, with varying integration of C_4_ plants (i.e. maize). We argue that, when compared to other datasets now available from elsewhere in the Amazon Basin, our study highlights the development of regional adaptations to local watercourses and forest types.

## Introduction

Archaeological and palaeoecological understanding of pre-colonial subsistence and population density in the Amazon Basin has undergone a major shift over the course of the last half a century [1]. In the second half of the 20^th^ century, ethnographic observations of small, hunting and gathering communities, as well as human ecological theories rooted in environmental determinism, were used to argue that poor soils, high humidity, and hydrological activity prevented the development of cultivation that could support large sedentary and complex societies in this part of the Neotropics [2–4]. Since then, however, archaeobotanical work has demonstrated that the tropical forests of the Amazon were a major center of crop domestication, including manioc (*Manihot esculenta* ssp. Esculenta), pineapple (*Ananas comosus*), rice (*Oryza* sp.) and peach palm (*Bactris gasipaes*) [5–7]. Meanwhile, studies have tracked the arrival of maize (*Zea mays*) from North and Central America into the region, and its integration into swidden cultivation strategies [8, 9]. Not only that, but there is evidence for the active management of freshwater resources [10], the movement and arboriculture of tree species such as the Brazil Nut (*Bertholletia excelsa*) [11], and burning to manage ecological structure and dynamics [12]. Pre-colonial Indigenous impacts are now known to have been so significant that they led to the widespread formation of Anthropogenic Dark Earth (ADE) soils and a lasting legacy on the species composition of tropical forests across the Amazon Basin that are still visible in the 21^st^ century [9, 13, 14]. Furthermore, mixed cultivation, fishing, and arboriculture seem to have sustained populations of up to 8–20 million at the time of European arrival [1, 15], and urban networks [16], earthworks [17], and other forms of landscape modification [10] have been documented across the basin.

Nevertheless, while the Amazon Basin has now been established as a key center of past human settlement and cultivation [15, 18–21], regional, multidisciplinary studies highlighting subsistence complexity are rare. As elsewhere in the Americas, there have been discussions of the importance of the expansion of maize in different parts of the Amazon Basin for the development of social stratification and complexity [22, 23]. However, detailed local studies have also highlighted human subsistence reliance on domesticated root crops such as manioc and non-domesticated plants and animals, including aquatic resources (e.g. [9, 24]). Indeed, given the vast area (7,500,000 km^2^) of the Amazon Basin and its forests, spanning eight countries, and a variety of different ecological zones, it is essential to develop more nuanced, contextual insights into variations in settlement distribution, geography, and human subsistence adaptations. This has traditionally been difficult, with warm, humid conditions often leading to poor preservation of organic plant and animal remains commonly used to reconstruct subsistence elsewhere [24]. Where such remains are preserved, supplemented by microbotanical approaches to studying plant use, it can still remain difficult to determine the degree to which human societies relied upon different food groups (e.g. [25]). This is significant when seeking to test hypotheses such as those which argues that differences between *terra firme* (upland habitats away from the floodplain) and *várzea* settings (seasonally flooded areas) would have dramatically shaped subsistence strategies, with the generally nutrient poor soils of the former necessitating use of forest resources or dramatic landscape modification, and the latter providing ample riverine products and rich soils for cultivation (e.g. [10, 26]). Similarly, while extensive archaeological sites are often associated with ADEs across the Amazon, there is remarkable diversity in pre-colonial ceramic typologies and funerary practices [27–34], though little is known about how this social and cultural diversity played out in terms of subsistence practices and economic adaptations.

**Fig 1:**
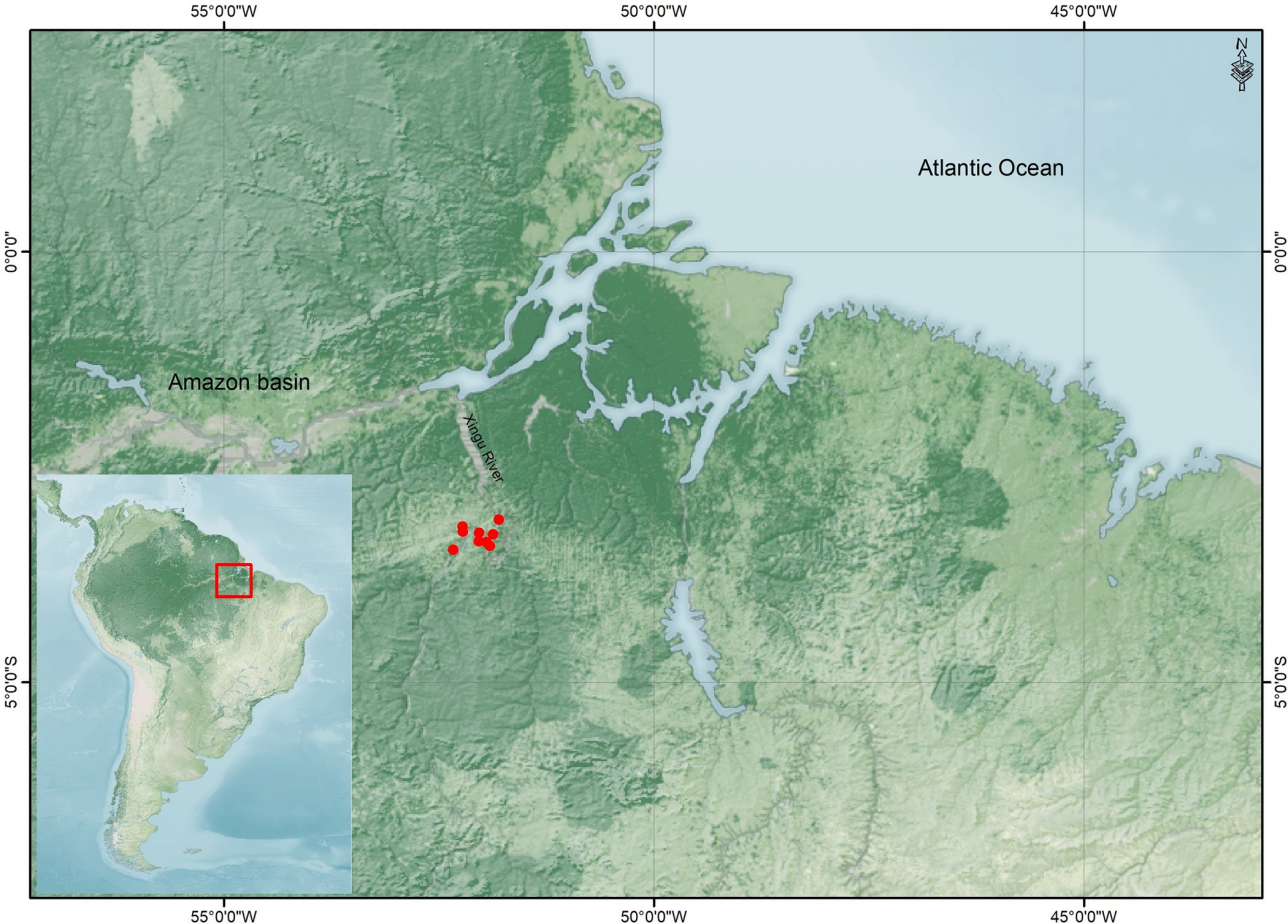
Map showing the location of the Amazon rainforest (darkest part), and archaeological sites of study in the Volta Grande do Rio Xingu (red points). The map was created for this study by Renato Gonzalez (Geoprocessing Analyst for the Scientia Consultoria Científica, Brazil) using QGIS 3.2.4 https://qgis.org/pt_BR/site/ and the Natural Earth Database from https://visibleearth.nasa.gov/images/147190/explorer-base-map (**Landsat images courtesy of NASA Goddard Space Flight Center and US Geological Survey)**.

Here, we undertake a detailed subsistence analysis of populations living at the Volta Grande do Rio Xingu (VGRX) between 390 cal. years BC and 1,675 cal. years AD (see Table 1) through the application of stable isotope analysis. The Upper Xingu is renowned for the discovery of so-called ‘Garden Cities’ that were fundamental in overturning ideas of the Amazon Basin as a ‘pristine wilderness’ [16, 35]. Although less known, archaeological research carried out in the middle and lower portions of the Xingu River have also uncovered an extremely rich, diverse and extended archaeological record [27, 28, 36–39], which in some cases presents well preserved organic remains. Despite its long history of application in archaeology, stable isotope analysis has rarely been applied across the vast area of the Amazon Basin [22, 40], although recent research at the Marajó Delta [24] and at the mouth of Mearim River (at the eastern limit of the Amazon Basin by the sea) [41] are highlighting its potential. In order to fill in gaps in the knowledge of dietary and cultural variability across this increasingly important region in the context of human-environment interactions, we apply stable isotope analysis to human and animal remains from twelve archaeological sites in the lower and middle portions Xingu River for the first time to explore the varying subsistence practices, such as degree of reliance upon wild and cultivated rainforest C_3_ plants, animal resources, and arriving C_4_ resources (i.e. maize), among groups with different cultural affiliations and micro-habitats within this part of the Amazon Basin (Fig 1).

**Table 1:** Radiocarbon analysis of archaeological sites from which isotope analysis were performed.

Site and ceramic association	Material	Level (cm)	Lab number	Results	δ^13^C	Cal 2α ^5^
Palhal 2 (Koriabo and Tupi)	Human Bone^1^	Burial 2	OxA-X-3050-26	424±25 BP	-16.8‰	1429 (92.8%) 1493calAD
						1602 (2.6%) 1611calAD
	Human Bone^1^	Burial 1	OxA-39692	390±19 BP	-17.4‰	1445 (81.5%) 1515calAD
						1598 (13.9%) 1618calAD
	Charcoal^1^	30–40	Beta-542851	520±30BP	-28.1‰	1324 (10.5%) 1345calAD
						1393 (84.9%) 1443calAD
	Charcoal^1^	60–70	OxA-33027	967±28 BP	-31.0‰	1018 (95.4%) 1155calAD
	Charcoal^1^	20–30	Beta-542854	370±30BP	-23.3‰	1485 (95.4%) 1650calAD
	Charcoal^1^	50–60	Beta-552222	350±30BP	-23.6‰	1458 (41.4%) 1531calAD
						1538 (54.1%) 1635calAD
Pimental 2 (Koriabo and Tupi)	Charcoal^1^	40–50	OxA-33028	248±26 BP	-26.9‰	1525 (7.5%) 1558calAD
						1631 (62.5%) 1675calAD
						1777 (22.3%) 1800calAD
						1941(3.1%) until now.
	Charcoal^1^	20–30	Beta-554247	680±30BP	-26.0‰	1324 (10.5%) 1345calAD
						1393 (84.9%) 1443calAD
Palmeiras (Koriabo and Tupi)	Human Bone^1^	Burial 1	OxA-X-3050-27	371±26 BP	-15.6‰	1270 (60.4%) 1316calAD
						1355 (35.0%) 1390calAD
	Human Bone^1^	Burial 2	OxA-X-3050-28	342±26 BP	-15.8‰	1470 (95.4%) 1637calAD
	Charcoal^1^	40–50	Beta-552227	810±30BP	-24.1‰	1169 (95.4%) 1270calAD
Pedra do Navio (Koriabo and Tupi)	Charcoal^2^	30–40	Beta-542864	610±30 BP	-27.0‰	1295 (95.4%) 1404calAD
	Charcoal^2^	60–70	Beta-542863	630±30BP	-24.9‰	1287 (95.4%) 1399calAD
Vila Rica 2 (Koriabo and Tupi)	Charcoal^3^	40–50	Beta-554251	850±30BP	-24.0‰	1052 (5.2%) 1080calAD
						1152 (90.2%) 1260calAD
São José 1 (Arauquinóide, Koriabo and Tupi)	Charcoal^1^	40–50	Beta-554248	2.240±30BP	-25.5‰	390 (25%) 345calBC
						323 (70.4%) 205calBC
Carrazedo (Koriabo)	Charcoal^4^	-	-	-	-	1.260–1.460cal AD
	Charcoal^4^	-	-	-	-	750–870 cal AD

^1^ This research.

^2^ Castro, 2020

^3^ Castro et al., 2021

^4^ Fernandes et al., 2018

^5^ Calibrated using OxCal v4.4.4 [94] on line with IntCal13 atmospheric curve [95]. The dates obtained by analysis of human bones were not corrected for the reservoir effect.

## Background

### Environmental and cultural contexts of the Xingu River

The Xingu River has its source in transitional environments between Amazonian evergreen rainforests and the Brazilian Cerrado and flows south-north into the Amazon River. It is a clear water river [42] and covers more than 1,900 km from its source to its mouth. The transition between the middle and lower reaches of the river is characterized by large rapids with a vertical difference of 85 m in the space of 160 km–the so-called Volta Grande of the Xingu River (VGRX). In the lower portion of the river, the slope is more gradual, resulting in larger floodplains that are more prone to flooding as a product of backwater effects from the Amazon River [43, 44]. The climate of the region is considered a ‘humid tropical micro-climate’ [45], with the average temperature of the coldest month being above 18°C. The vegetation along the middle and lower reaches of the Xingu River is characterized by dense ombrophylous forest [46]. Recent studies on the fauna and flora of the middle and lower reaches of the Xingu River point to the presence of more than 440 species of birds, 259 mammals–including taxa regularly reported as food sources used by Indigenous populations such as deer (*Mazama nemorivaga* and *Mazama americana*), tapir (*Tapirus terrestris*), paca (*Cuniculus paca*) and agouti (*Dasyprocta aguti*), as well as primates, giant otters (*Pteronura brasiliensis*) and otters (Lutrinae)– 174 reptile species including alligators (*Melanosuchus niger*), tortoise (*Chelonoidis* sp.*)* and tracajá (*Podocnemis Unifilis*), and rich ichthyofauna populations (fish) frequently consumed by humans [47]. Within this ecological context, the rapids of the Volta Grande act as a geological barrier to certain taxa such as porpoises, manatees and important fish such as pirarucu (*Arapaima gigas*) and dourada (*Salminus brasiliensis*), which are not found in the portion above the waterfall [47]. Available tree species, such as Brazil Nut (*Bertholletia excelsa*), babassu (*Attalea speciosa*), açai-palm (*Euterpe oleracea*) and a wide variety of other fruit trees, are known to be favoured by Indigenous hunter-gatherers [19, 48].

Anthropological and ethnohistorical studies show that a diversity of Indigenous communities have lived, and still live, in the middle and lower reaches of the river [49, 50], speaking languages belonging to one of three groups–Tupiguarani, Aruak and Jê [51, 52]. Recent archaeological surveys [29, 38, 53] indicate that there was an increase in population density along the middle and lower Xingu River around 1,500 cal years BP, with large archaeological sites, increased densities of material culture and ADEs occurring at this time. The recovered ceramics from archaeological sites dated to this period indicate significant regional and temporal diversity, with pottery often associated with Koriabo, Arauquinoid (both related to Karib speakers), and Tupiguarani (related to Tupiguarani speakers) industries [54, 55]. Koriabo ceramics are traditionally found in the state of Amapá (Brazil’s State) [56] and Guianas [57], and their presence at the mouth of the Xingu River [28], middle Xingu River [38, 58], and lower Amazonas [59] has led to hypotheses of population movements from the Guianas into the Brazilian Amazon. The same is true of Arauquinoid ceramics, initially identified in Venezuela and French Guiana, but noticed in different archaeological sites along the Amazon River [60]. Tupiguarani ceramics are found in many archaeological sites in the interfluvial area between the Tocantins and Xingu rivers, displaying a well-established temporal sequence, beginning in the third century AD [61, 62].

Ethnohistorical studies of accounts of the earliest travelers into the region (17^th^-20^th^ centuries) suggest that the majority of Indigenous groups living along the middle and lower Xingu River had subsistence strategies based on the cultivation of manioc, maize or sweet potato [51, 63–67]. Nevertheless, no archaeobotanical remains of these crops have been found at any of the excavated sites in the region, although no microbotanical analysis has yet been performed. Ethnohistorical accounts also indicate varying reliance on wild fauna, fish and plant resources [51, 63–68]. The preliminary results of faunal analyses indicate the presence of many faunal taxa at ADE sites, including tapir (*Tapirus* sp.), paca (*Cuniculus paca*), agouti (*Dasyprocta* sp.), capybara (*Hydrochoerus hydrochaeris*), Brazilian rabbit (*Silvilagus brasiliensis*), monkeys (Primatas), deer (Cervidae), turtles (Testudines), alligators (Alligatoridae), nine-banded armadillo (*Dasypus novemcinctus*), sloth (*Bradypus* sp.) and fish such as the Vampire Fish/Cachorra (*Hydrolycus scomberoides*). Some of these remains have cut marks and signs of fire exposure. In the context of wider Amazon Basin subsistence questions, of particular interest is the spread of maize (known to be present on the Upper Madeira River back to 6,500 cal. BP and at the mouth of the Amazon from 4,300 cal. BP) [8, 9, 69], as well as the types of subsistence strategies that supported growing populations, including the ‘Garden Cities’, found in upper Xingu River from 1,000 cal AD [35], and dietary variability between individuals associated with pottery of different cultural affiliations.

### Isotope analysis in the tropics

Bioarchaeological studies that can directly assess the varied subsistence practices of past Xingu River populations are sorely needed. Stable isotope analysis has been used for paleodietary reconstruction in archaeology since the 1970s [70]. Stable carbon isotope (δ^13^C) variability in tropical terrestrial ecosystems is primarily driven by plants that utilize the two different dominant photosynthetic pathways, C_3_ and C_4_ [71]. In C_3_ plants, strong discrimination against ^13^C during CO_2_ fixation results in lower δ^13^C values in the vast majority of trees and shrubs which dominate tropical forest environments, as well as domesticates such as manioc, relative to wild C_4_ grasses or domesticates such as maize [72]. C_3_ δ^13^C values vary from *c*. -24 to -36‰ (global mean -26.5‰), while C_4_ values range from *c*. -9 to -17‰ (global mean -12‰) [71]. These distinctions are reflected in the tissues of consumers eating these plants, with small trophic level effects of 1–2‰ [73]. Within a C_3_-dominated context, further variation occurs due to the ‘canopy effect’ which results in C_3_ plants living under a dense canopy having lower δ^13^C than those living in more open environments [74].

δ^15^N in terrestrial ecosystems varies with trophic level, and δ^15^N trophic shifts of between +2–6‰ have been documented in terrestrial and aquatic systems [75, 76]. The long length of marine food chains, leads to distinctively high δ^15^N in marine organisms [77]. Freshwater organisms tend to also have high δ^15^N for the same reason. However, freshwater δ^13^C does not follow the same co-varying trend with δ^15^N towards higher measurements that is seen in marine food chains due to different sources of carbon in these environments, and highly variable δ^13^C has been observed in freshwater settings around the world [78]. These principles and distinctions make stable isotope analysis a highly powerful methodology for testing a number of important questions about past human diets. However, although thresholds of 100% C_3_ consumption, 100% C_4_ consumption, or ‘canopy consumption’ have been established, environmental factors [79], which can lead to both δ^13^C and δ^15^N variability as a result of soil dynamics and climatic effects (e.g. rainfall), mean that it is essential to generate baseline data from associated animal remains in archaeological sites.

δ^13^C and δ^15^N analysis of human bone collagen (δ^13^C_co_) primarily determines the isotopic values of the *protein* input to the diet, with a much more minor contribution of lipid and carbohydrate sources [73]. This means that the δ^13^C_co_ and δ^15^N values of bone collagen will be heavily affected by foods that are high in protein, such as fish and meat [73]. By contrast, δ^13^C measurements of tooth enamel bioapatite (δ^13^C_ap_) reflect the ‘whole-diet’ during the period of enamel formation that will vary depending on species and tooth sampled [80]. These tissues will also represent different periods of diet depending on the element sampled. Femur bones have been argued to reflect the last 10 years of life. Meanwhile, tooth enamel will reflect different periods depending on the formation of the tooth sampled, with M3s representing the latest period of childhood (c. 9–11 years) [81].

## Materials and methods

### Archaeological sites

The human material analyzed comes from ten open-air archaeological sites located along the middle Xingu river (VGRX) and one along the lower Xingu river where it flows into the Amazon river (site descriptions are found in S1 File). The faunal remains come from seven archaeological sites located at VGRX and one from the lower Xingu River site (Table B in S1 File). With the exception of the Bela Vista and Carrazedo sites, the archaeological sites were excavated between 2011 and 2015 as part of the environmental licensing process for the Belo Monte hydroelectric dam, located in the middle Xingu River. The Bela Vista site was preliminarily studied by a team from the Museu Paraense Emílio Goeldi (MPEG) through a rescue excavation, when an urn was exhumed [43]. In 2018, its internal contents were excavated in laboratory [82]. The Carrazedo site has been excavated by the MPEG team since 2014 as part of an international project called OCA [28, 83]. With the exception of one site from the middle Xingu (Santo Antônio 1) [84], all of the studied sites presented ADE soils, and organic materials, such as faunal and human remains, found in association with these ADEs (see more descriptions in S1 File).

Existing radiocarbon archaeological assays from VGRX indicate a long and intensive occupation of the region by human societies for at least 10,000 years, initially represented by foraging groups [85]. Populations associated with simple ceramic forms are found from 4.000 cal. years BP, prior to a period of demographic and socio-cultural diversification around 1.500 cal. years BP, reflected in the presence of Arauquinoid, Koriabo, Tupiguarani ceramic styles and some influences from the Upper Xingu style [29, 58]. Occasionally, some of these styles are present in the same occupation layers of the same site and the same burial contexts [38, 58]. Human burial patterns are also diverse, both in their form and in terms of grave goods. Two or more burial practices may occur in the same place (see more descriptions in S1 File), as primary burials without funerary accompaniments, primary burials accompanied by Tupiguarani and Koriabo style vessels, and burials within urns. To make the chronology of occupation of these sites more robust, samples of charcoal (Palhal 2, Pimental 2, Palmeiras and São José 1 sites), human bones (Palhal 2 and Palmeiras sites) and fauna (Palhal 2 site) were sent for ^14^C analysis at the Beta Analytic Inc Laboratory and the Research Laboratory for Archeology and History of Art (RLAHA), University of Oxford (the method of analysis and details of samples analyzed at these laboratories can be found in Table A in the S1 File).

### Samples

Research permissions were issued by the Instituto do Patrimônio Histórico e Artístico Nacional (Iphan, Brazil), through process n01492.000507/2018-79. We analyzed human bones and teeth from 26 single burials from the sites discussed above, representing all of the available human individuals for sampling. Of the 26 individuals investigated, 13 had both bone and teeth samples analyzed, nine had only bones, and four had only teeth (Table C in S1 File). For stable isotope analysis of bone collagen, femurs and tibias were preferably sought as representative of the longest period of life [86]. However, skull fragments (parietals or occipitals bones) were used where they were the only bone elements available. When present, the 2^nd^ and 3^rd^ molars were sampled for isotope analysis of human tooth enamel, as they represent the latest period of diet (c. 4–11 years of age) available from this biogenic material [87]. Where not available, we sampled the teeth that were present in a given burial context. Some of these will potentially be impacted by the ‘weaning effect’ which has been argued to alter enamel δ^13^C by, on average, 0.5‰ [88]. However, this is unlikely to impact broader interpretations of resource use (Fig A in S1 File).

In order to reconstruct an isotopic baseline to aid interpretation of the data from the human remains, we analyzed 176 faunal bones and 28 teeth from the archaeological sites mentioned above, representing the available faunal material with clear taxonomic identification and good preservation (Table D in S1 File). Seven of these sites are situated in the VGRX and one, from the Carrazedo site, at the mouth of the Xingu, meaning that these remains act as suitable comparison for the human remains sampled. Sampling was primarily based on the material preserved and it was not possible to follow strict selection criteria (e.g. minimum number of individuals per species or a particular bone from the same anatomical region of all individuals). Instead, the samples reflect what was randomly preserved at archaeological sites that can be directly related to contexts with human remains. The fauna sampled include fish (Osteichthyes) such as Vampire tetra fish (*Hydrolycus scomberoides)* and Pacu fish (Myleinae); small and medium herbivorous mammals, such as deer (*Mazama* sp.), Lowland paca (*Cuniculus paca)*, Tapir (*Tapirus* sp.), Agouti (*Dasyprocta* sp.), Capybara (*Hydrochoerus hydrochaeris*), Collared peccary (*Pecari tajacu*), and White-lipped peccary (*Tayassu pecari*); omnivorous mammals such as the Nine-banded armadillo (*Dasypus novemcinctus*),; reptiles such as Tortoise (Chelonoidis), Turtle (Testudinata), Alligator (Alligatoridae); and birds (See S1 File for more information about fauna behaviour in the Amazon). The fauna were identified by Mariane Ferreira and Dr. Renato Kipnis (Scientia Consultoria Científica, Brazil) using the reference collections from Scientia Consultoria Científica and the University of São Paulo (Laboratório de Estudos Evolutivos Humanos do Instituto de Biociências -LEEH-IB- and Laboratório de Zooarqueologia e Bioarqueologia—LABZB). Broad identifications were often necessary due to the poor preservation of these specimens.

The human and faunal material from the Bela Vista and Carrazedo sites was sampled at MPEG in the year 2018. The human and faunal material from the Pedra do Navio, Gaioso 13, Vila Rica 2, Palmeiras, Palhal 2, Santo Antônio 1, São José 1, Santa Luzia 1, and Pimental 2 sites was sampled in the laboratory of Scientia Consultoria Científica, in Belém (PA), also in 2018.

### Stable isotope analysis

Bone collagen was extracted from a range of skeletal elements following standard procedures [89]. Although humic acids have been shown to potentially represent a contamination problem in tropical conditions, no corresponding staining of the bone material was identified here. As a result, we followed a similar extraction protocol to that undertaken by Hermenegildo et al. [24] in the Amazon Basin in order to ensure that our data is comparable (cf. [90]). Future work is needed to explore the exact impact of humic acids on bone preservation in the Amazon Basin (see also [91]). In this study, we employ the usual standard protocol for evaluating collagen preservation and quality.

Approximately 1.5 grams of bone, cleaned using sand abrasion, was demineralized in 10 ml aliquots of 0.5M HCl at 4°C. Acid was changed every 48 hours until the bone was fully demineralized. Demineralization took 3–20 days depending on the sample. The sample was then rinsed three times in deionized water before being gelatinized in pH3 HCl at 70°C for 48 hours. The resulting solution was filtered using an EZEE filter, and frozen overnight. The samples were then lyophilized over a period of 24 hours or until completely dry. After calculating the collagen yield, 1mg of extracted collagen sample was measured into a tin capsule to be analyzed for δ^13^C and δ^15^N in duplicate at the Stable Isotope Research Laboratory of the Department of Archaeology, Max Planck Institute for the Science of Human History using the Thermo Fisher Elemental Analyzer coupled to a Thermo Fisher Delta V Advantage Isotope Ratio Mass Spectrometer via a ConFloIV system. Two-point calibrations were performed using measurements of international standard reference materials (USGS40 L-Glutamic Acid: δ^13^C_raw_ = -26.4‰±0.1‰, v^13^C_true_ = -26.4‰±0.0‰, δ^15^N_raw_ = -4.4‰±0.1‰, δ^15^N_true_ = -4.5‰±0.1‰; IAEA N2 δ^15^N_raw_ = 20.2‰±0.1‰, δ^15^N_true_ = 20.3‰±0.2‰; IAEA C6 δ^13^C_raw_ = -10.9‰±0.1‰, δ^13^C_true_ = -10.5‰±0.0‰) with each analytical run.

Teeth or tooth fragments were cleaned using sand abrasion to remove adhering external material. 8 mg of enamel powder was obtained using gentle abrasion with a diamond-tipped drill along the full length of the buccal surface or fragment in order to maximize the period of formation represented by the sample. Enamel powder was pre-treated using a protocol to remove any organic or secondary carbonate contaminates [92, 93]. This consisted of the application of 1% sodium hypochlorite for 60 minutes, followed by three rinses in ultra-pure H_2_O, with vortexing and centrifuging, after each rinse, before 0.1M acetic acid was added for 10 minutes, followed by another three rinses in ultra-pure H_2_O, vortexing and centrifuging [80, 93]. Samples were then frozen and freeze dried for 4 hours. Approximately 2.5 mg of the treated sample were weighed into borosilicate glass vials and capped. The vials were flush/filled with helium at 100ml/min for 10-min and 20ul of phosporic acid was added. Following reaction with 100% phosphoric acid, gases evolved from the samples were analyzed for δ^13^C and δ^18^O using a Thermo Gas Bench 2 connected to a Thermo Delta V Advantage Mass Spectrometer at Stable Isotope Research Laboratory of the Department of Archaeology, Max Planck Institute for the Science of Human History. Obtained δ^13^C and δ^18^O isotope values were calibrated against international standards (IAEA NBS 18: δ^13^C -5.014 ± 0.032 ‰, δ^18^O -23.2±0.1 ‰, IAEA 603: δ^13^C +2.46±0.01 ‰, δ^18^O -2.37±0.04 ‰, IAEA CO8: δ^13^C -5.764±0.032‰, δ^18^O -22.7±0.2 ‰, USGS44: δ^13^C = ~ -42.1 ‰) registered by the International Atomic Energy Agency. Replicate analyses of standards suggest that machine measurement error is c. ± 0.1‰ for δ^13^C and ± 0.2‰ for δ^18^O. Overall measurement precision was studied through the measurement of repeat extracts from a bovid tooth enamel standard (n  =  20, ± 0.2‰ for δ^13^C and ± 0.4‰ for δ^18^O).

### Statistical analysis

All isotopic datasets were tested for normality using the Shapiro Wilk test. Following this test, the significance of the variation of δ^15^N and δ^18^O between dietary groups within the faunal datasests from VGRX (i.e. omnivores, carnivores, herbivores and fish) was determined using comparative ANOVA tests. An ANOVA test was also applied to compare the values of δ^15^N found for the fauna from VGRX with the values obtained in previous studies for archaeological samples from Ucayali Basin, in Peruvian by taxa [40]. In both cases, where variance was found to be significant, this was combined with a post-hoc Tukey pair-wise comparison test to determine which taxa or dietary groups were significantly different from each other.

The significance of δ^13^C_co_ and δ^13^C_ap_ variation between dietary groups of fauna at VGRX was determined using Kruskal-Wallis comparative tests, followed by Pairwise comparisons using Wilcoxon comparisons. The same approach was used to compare δ^13^C_ap_ values between fauna taxa from VGRX and values obtained in previous studies for archaeological and modern samples from the Peruvian Amazon [40, 79], and to compare δ^13^C_co_ values between fauna taxa from VGRX and values obtained in previous studies for archaeological samples from Ucayali Basin, in Peru [40]. Kruskal-Wallis tests were also used to compare the δ^13^C_co_ and δ^15^N values of the VGRX human groups with other groups previously studied (Ucayali Basin, Orinoco Basin, Lower Amazonas Basin and São Luis). The complete statistical results tables can be found in the S1 File (Tables I-U in S1 File). All statistical analyses were conducted using the free program R software (R Core Team, 2013).

## Results

### Radiocarbon dating

**Fig 2:**
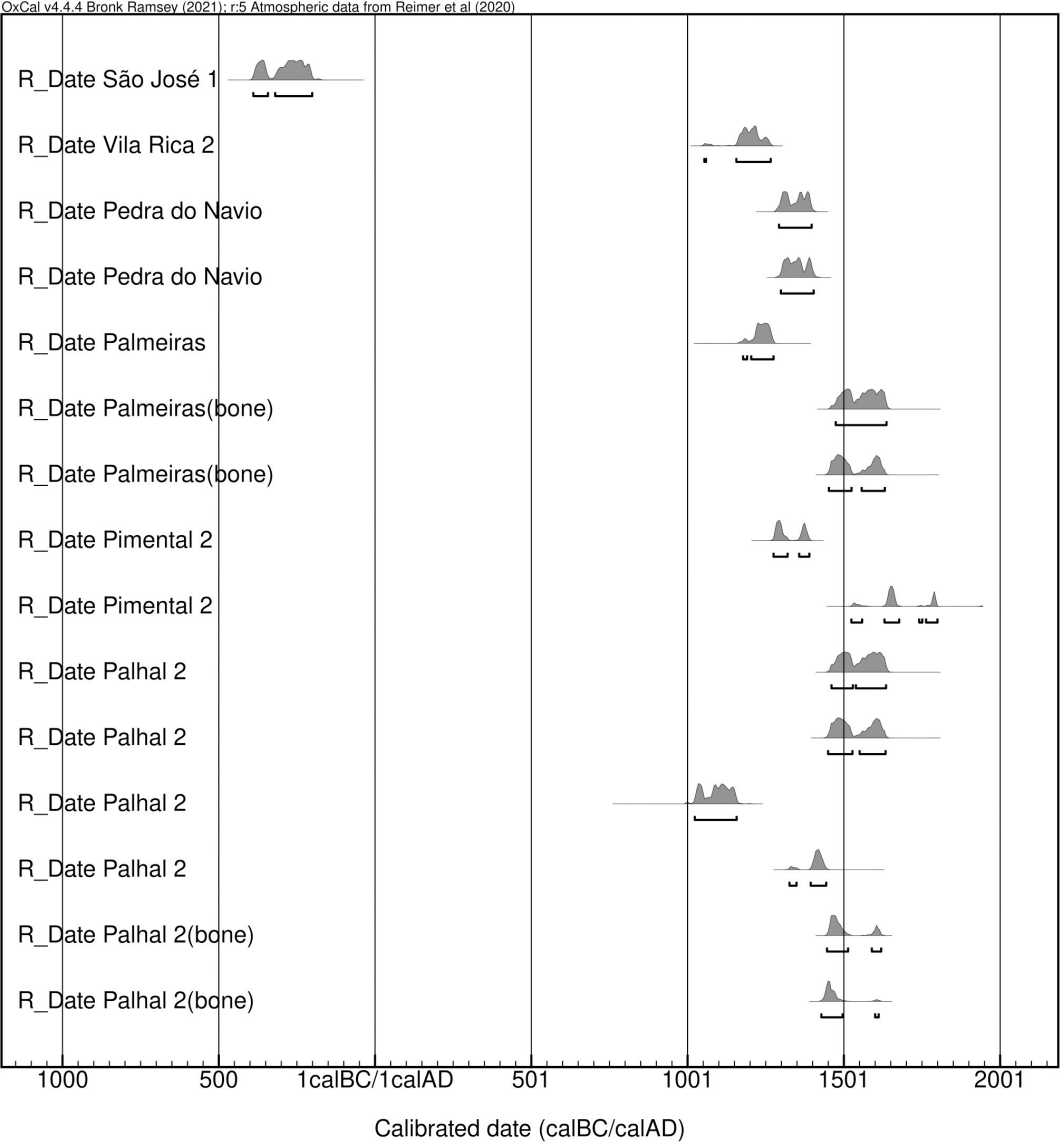
Dates from archaeological sites from VGRX. Most were occupied in the last millennium. Calibrations performed online using OxCal 4.4.4.

From the 11 sites studied in this research, previously published dates come from the Carrazedo, Vila Rica 2 and the Pedra do Navio sites. Carrazedo is located at the mouth of the Xingu River and has two dates that place the occupation of the site between 750–870 cal AD and 1260–1460 cal. AD [36]. Vila Rica 2, located at the VGRX, has one date that places the occupation of the site between 1052–1260 cal. AD [38], and Pedra do Navio, also located at VGRX, has two dates, placing the occupation of the site between 1295–1399 cal. AD [58]. Here, we present new ^14^C dates, using charcoal samples and human bone collagen, from a further four of the 11 sites studied in this research. These dates have not yet been published and provide a chronological picture of occupations at VGRX over the past two millennia (Table 1). The radiocarbon dates show a general focus of site occupations between 1,050 cal AD and 1,650 cal AD, with one displaying greater antiquity, 300 cal BC, possibly reflecting the beginning of the occupation of that archaeological site (Table 1, Fig 2) (See in S1 File more information). The São José 1 site, from which the oldest date comes, is also the only site sampled with Arauquinoid type ceramics, with which the four exhumed individuals are possibly associated.

### δ^13^C_co_ and δ^15^N from collagen

The poor preservation of organic materials within tropical forests is well-attested (e.g. [96]), and this study has faced some issues in this regard. For the analysis of bone collagen, of the 22 human individuals from which small portions of bone were analyzed, only four adults from two archaeological sites had δ^13^C_co_ and δ^15^N values from collagen with acceptable C/N ratios (between 2.9 and 3.6) and collagen yields (>1%) [97, 98] (Table 2) (Table V in S1 File). The δ^13^C_co_ values of the two individuals from the Palmeiras site are very close to each other with values of -16.3‰ and -15.7‰ (dated to 371±26 BP and 342±26 BP, respectively). These values are also similar to the δ^13^C_co_ value obtained from an individual from Palhal 2 site (-16.0‰), though higher than that of a second individual from the same site (-18.2‰) (dated 390±-196 BP and 424±24 BP, respectively). The δ^15^N values for the four individuals varied by up to 1.5‰, being slightly higher in individuals from the Palhal 2 site (13.2‰ and 12.4‰) than in the Palmeiras site (11.3‰ and 11.5‰).

**Table 2:** Human stable isotopes results from collagen.

Site	Burial	Lab Cod	δ^13^C_co_ ‰VPDB	%C	δ^15^N ‰AIR	%N	Ratio C/N	Sex
Palmeiras 1	Burial 1	PAM-001	-16.3	25.0	11.3	8.9	3.30	Male
Palmeiras 1	Burial 2	PAM-002	-15.7	44.6	11.5	17.6	3.50	Male
Palhal 2	Burial 1	PAL-001	-18.2	36.5	13.2	13.4	3.20	Male
Palhal 2	Burial 2	PAL-002	-16.0	44.8	12.4	18.1	2.90	Male

**Fig 3:**
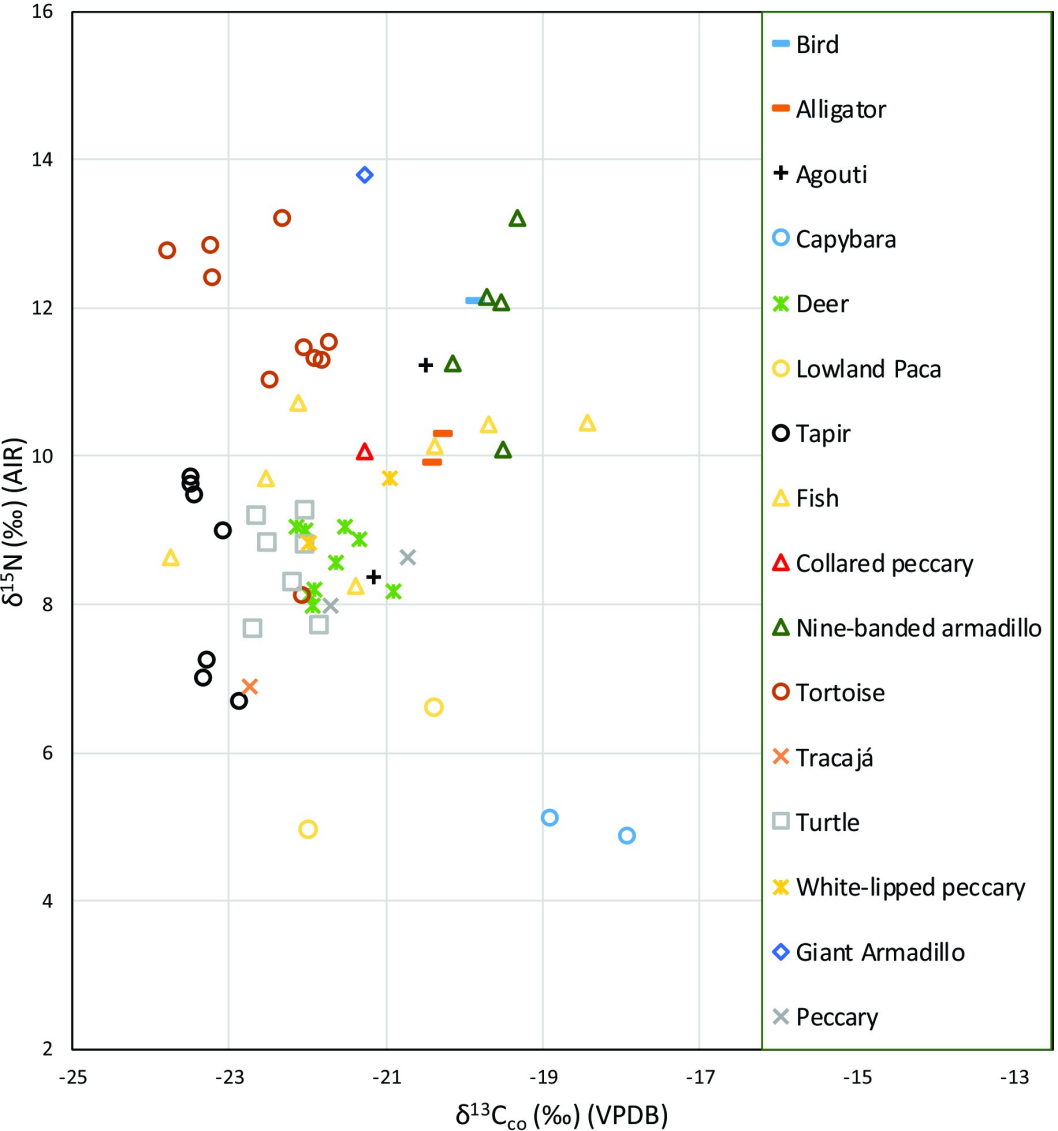
Fauna bulk bone collagen δ^13^C and δ^15^N results for VGRX.

**Fig 4:**
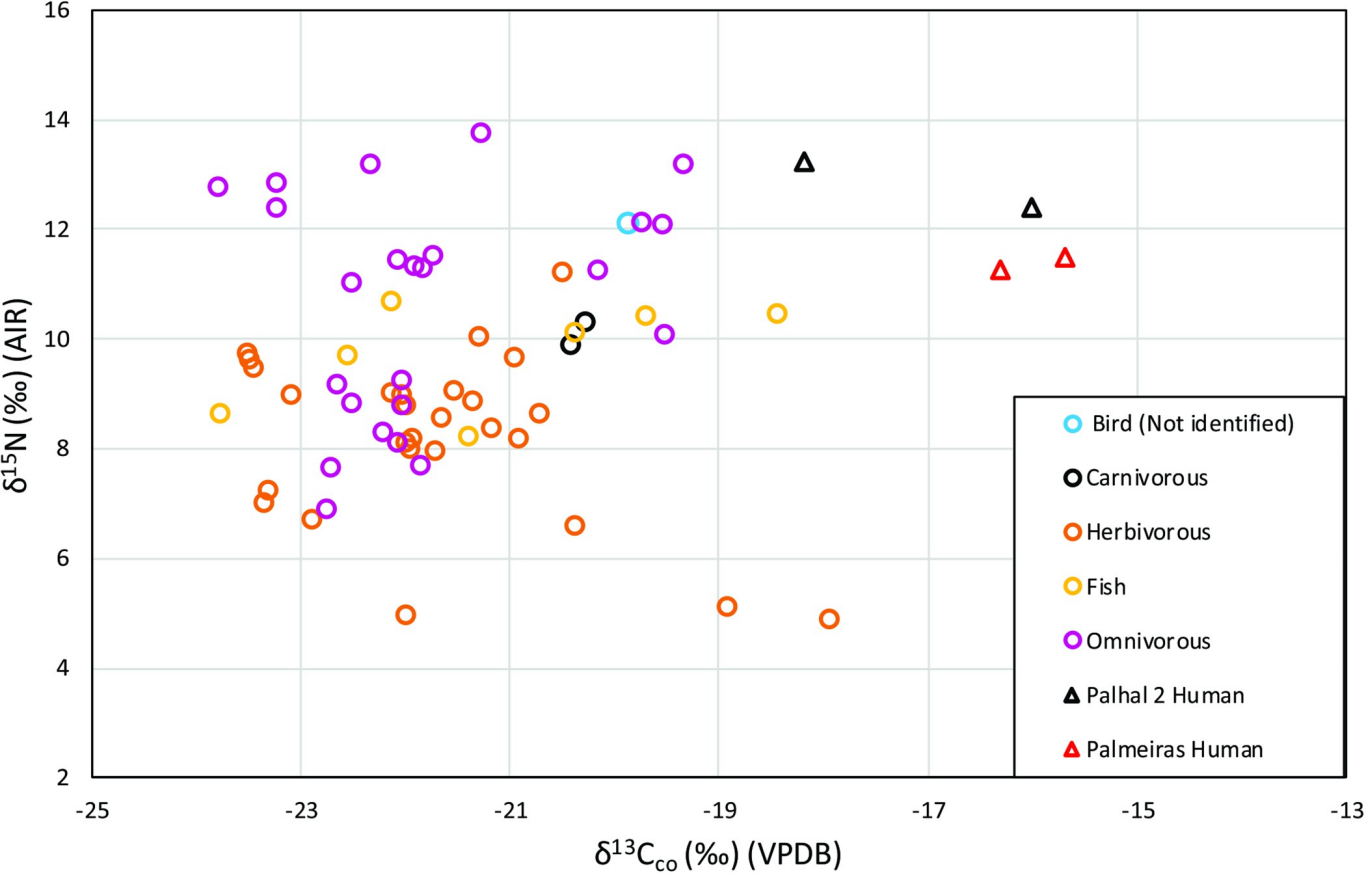
Fauna and human bulk bone collagen δ^13^C and δ^15^N results grouped by diet for VGRX.

Low preservation was also observed for the collagen of the faunal bones analyzed, and, of the 176 samples analyzed, only 61 presented satisfactory C/N ratios and collagen yields (Table E in S1 File). The analyzed terrestrial fauna are composed of a wide variety of herbivorous taxa (capybara, paca, tapir, agouti, peccary, and deer) and represent the largest dataset of pre-industrial δ^13^C_co_ isotopic characterization for an Amazonian faunal community to date (Figs 3 and 4). The average δ^13^C_co_ and δ^15^N values for herbivores are -21.7‰±1.3‰ and 8.2‰±1.6‰, respectively (*n* = 27, range = -23.5‰ to -17.9‰ for δ^13^C_co_ and 4.9‰ to 11.2‰ for δ^15^N). Among the sampled omnivores (tortoises, turtles, tracajá, and armadillos) the average values of δ^13^C_co_ and δ^15^N are -21.8‰±1.3‰ and 10.6‰±2.1‰, respectively (*n* = 24, range = -23.8‰ to -19.3‰ for δ^13^C_co_ and 6.9‰ to 13.8‰ for δ^15^N). Among carnivores (alligator) the mean values of δ^13^C_co_ and δ^15^N are -20.3‰±0.1‰ and 10.1‰±0.3‰, respectively (*n* = 2, range = -20.4‰ to -20.3‰ for δ^13^C_co_ and 9.9‰ to 10.3‰ for δ^15^N). These values fall very close to those obtained for a non-identified bird, which presented a δ^13^C_co_ of -19.7‰ and δ^15^N of 12.1‰.

Freshwater fish resources, rich in species diversity and widely consumed by past and present Amazonian populations, were identified in the archaeological record to the level of class. The seven specimens analyzed from the archaeological contexts had average δ^13^C_co_ and δ^15^N values of -21.2‰±1.8‰ and 9.8‰±1.0‰, respectively (*n* = 7, range = -23.8‰ to -18.5‰ for δ^13^C_co_ and 8.3‰ to 10.7‰ for δ^15^N). This large variation in the values of δ^13^C_co_ is evident among individuals of the same species and for different species and highlights the isotopic unpredictability of freshwater ecosystems, especially those as ecologically diverse as those of the Amazon Basin [78]. While different dietary sources also contribute to δ^13^C_co_, variation in the values of δ^15^N may be related to trophic level [77] as well as the size and age of the fish sampled. Using Kruskal-Wallis statistical testing, we found no significant differences between the δ^13^C_co_ values of herbivores, carnivores, omnivores and fish in the VGRX sample (chi-squared = 3.2098, df = 3, p-value > 0.05) (Table I in S1 File). ANOVA and post-hoc Tukey pair-wise comparison showed that for δ^15^N, herbivores were statistically different from fish (*p*<0.003) and omnivorous taxa (*p* = 0.000), but did not statistically differ from carnivorous taxa (represented by only two alligators) (*p* = 0.089). There are no statistically significant δ^15^N differences between fish, omnivores, and carnivores (Table K in S1 File).

### δ^13^C_ca_ and δ^18^O from enamel bioapatite

Tooth enamel bioapatite differs from bone collagen and dentine in that it is more resistant to post-mortem replacement and diagenetic degradation [99, 100]. For the analysis of tooth enamel stable isotope measurements, all of the 17 human subjects, from nine different archaeological sites, who had small portions of teeth analyzed, presented results (Table 3) (Table F in S1 File). When observing the results of human δ^13^C_ap_ from VGRX a relatively large range of values is observed, including between individuals from the same site. The average human value of δ^13^C_ap_ was -10.4‰±1.3‰ (range = -13.3‰ and -8.4‰). δ^18^O values, meanwhile, presented an average of -3.2‰±0.6‰ and a range of -4.4‰ to -2.0‰.

**Table 3:** δ^13^C_ap_ and δ^18^O values for enamel from prehistoric human from VGRX, archeological sites in the Xingu River Basin, Brazil.

Archaeological site	Burial	Lab Cod	Tooth	δ13C (‰) (VPDB)	Std. dev.	δ18O (‰) (VPDB)	Std. dev.	Sex
Bela Vista	Urn 1	BEL-001	M	-8.4	0.3	-2.0	0.2	Not identified
Gaioso 13	Structure 1	GAI-001	M	-11.0	0.1	-3.7	0.1	Not identified
Palhal 2	Burial 2	PAL-002	3RLM	-11.9	0.1	-2.9	0.1	Male
Palmeiras 1	Burial 1	PAM-001	3RLM	-11.2	0.2	-3.0	0.1	Male
Palmeiras 1	Burial 2	PAM-002	2LUM	-8.7	0.2	-3.1	0.1	Male
Pedra do Navio	Urn 1	PED-001	1LLPM	-10.3	0.1	-4.4	0.1	Not identified
Pedra do Navio	Burial 1 (structure 4)	PED-002	2LLPM	-8.8	0.2	-2.9	0.1	Not identified
Pedra do Navio	Burial 2 (structure 6)	PED-003	1LRPM	-10.6	0.2	-2.8	0.1	Female
Pimental 2	Burial 2	PIM-002	M	-10.0	0.2	-2.8	0.1	Female
Pimental 2	Burial 4	PIM-004	1LLM	-10.0	0.2	-3.0	0.1	Not identified
Pimental 2	Urn 2	PIM-007	M	-10.4	0.2	-4.0	0.1	Not identified
Santo Antônio 1	Urn 2	SAN-001	M	-12.1	0.2	-3.4	0.2	Not identified
São José 1	Burial 2	SÃO-002	1RUM	-11.4	0.1	-4.1	0.1	Male
São José 1	Burial 3	SÃO-003	M	-13.3	0.1	-3.1	0.1	Not identified
São José 1	Burial 4	SÃO-004	PM	-10.6	0.2	-3.2	0.1	Female
Vila Rica 2	Structure 28	VIL-001	2LM	-10.2	0.6	-2.9	0.2	Not identified
Vila Rica 2	Structure 29	VIL-002	LPM	-8.8	0.2	-2.6	0.1	Not identified

The individual with the lowest δ^13^C_ap_ came from the São José 1 site (δ^13^C_ap_ -13.3 ‰), the site with the oldest radiocarbon date (2,240 ± 30 BP). The other two individuals from the São José 1 site, had δ^13^C_ap_ values of -11.4 ‰ and -10.6 ‰, that are similar to those seen in other sites such as Gaioso 13 (-11.0 ‰), Palhal 2 (-11.9 ‰, bone from the same individual was dated to 390±19 BP), Pimental 2 (-10.0 ‰, -10.0 ‰ and -10.4 ‰, the site was dated to 248 ± 26 BP and 680 ± 30 BP), one individual from Palmeiras 1 (-1.2 ‰, bone from the same individual dated to 371±26 BP), two individuals from Pedra do Navio (-10.3 ‰ and -10.6 ‰, site dated by radiocarbon to 610 ± 30 BP and 630 ± 30 BP), and an individual from Vila Rica 2 (-10.2 ‰, site dated by radiocarbon on charcoal to 850 ± 30 BP). The highest human δ^13^C_ap_ value belonged to the individual from the Bela Vista site (δ^13^C_ap_ -8.4 ‰). Although this archaeological site has yet to yield an absolute date, its material culture is similar to that of other sites in the region dated to the last millennium. Other humans with similar values come from the Palmeiras site (-8.6 ‰, bone from the same individual dated to 342±26 BP) and one individual from Pedra do Navio (-8.8 ‰).

**Fig 5:**
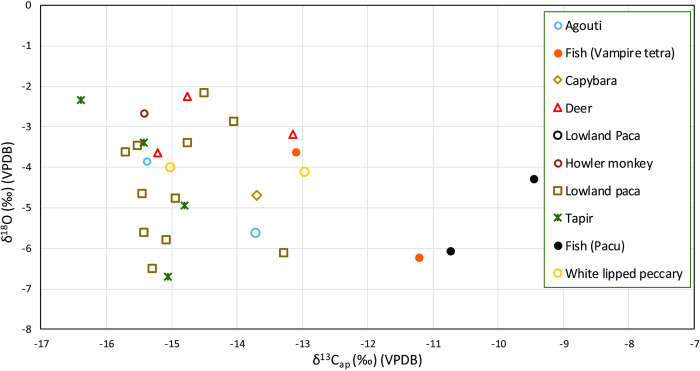
Faunal bulk tooth enamel δ^13^C and δ^18^O for VGRX.

**Fig 6:**
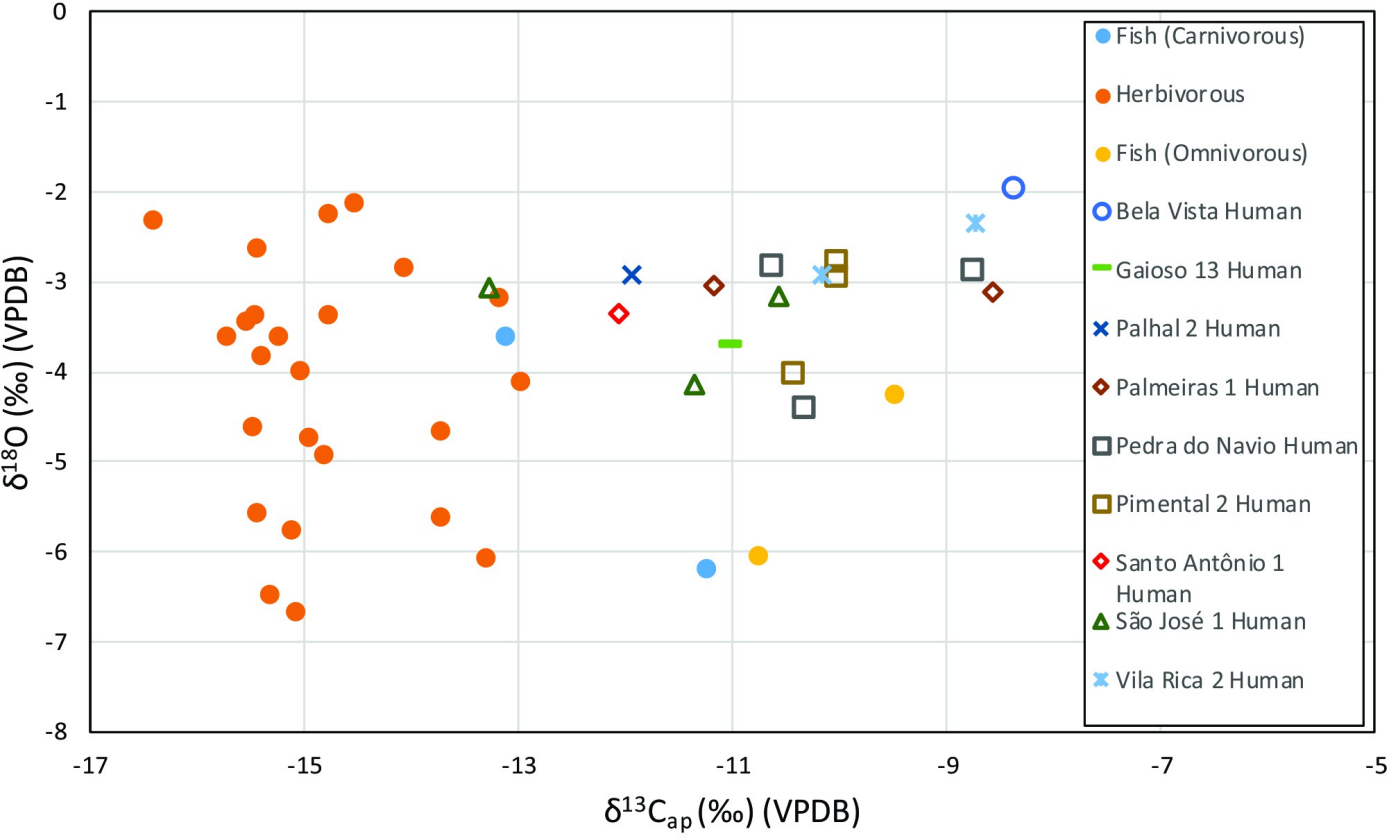
Faunal and human bulk tooth enamel δ^13^C and δ^18^O grouped by diet for VGRX.

The tooth enamel samples from terrestrial and aquatic fauna comprise 28 individuals that can be grouped into herbivorous and fish taxa [101] (Table G in S1 File) (Figs 5 and 6). The herbivorous average values of δ^13^C_ap_ and δ^18^O are -14.8‰±0.9‰ and -4.2‰±1.4‰, respectively (*n* = 24, range = -16.4‰ to -13.0‰, and -6.7‰ and -2.2‰ for δ^18^O). Two types of fish had their dental enamel analyzed, one with an omnivorous diet (Myleinae, *n* = 2) and one with a carnivorous diet (*Hydrolycus scomberoides*, *n* = 2). The omnivorous specimens had average δ^13^C_ap_ and δ^18^O values of -10.1‰±0.9‰ and -5.2‰±1.3‰, respectively (*n* = 2, range = -10.7‰ to -9.5‰ for δ^13^C_ap_ and -6.1‰ and -4.3‰ for δ^18^O). The carnivorous specimens had average δ^13^C_ap_ and δ^18^O values of -12.2‰±1.3‰ and -4.9‰±1.8‰, respectively (*n* = 2, range = -13.1‰ to -11.2‰ for δ^13^C_ap_ and -6.2‰ and -3.6‰ for δ^18^O). We found no significant differences between the δ^18^O values by dietary group in the VGRX sample (Tables M and N in S1 File). A Kruskal-Wallis test confirms that there is a difference in δ^13^C_ap_ by diet (chi-squared = 9.6218, df = 2, p-value < 0.05) but a Wilcoxon Pairwise comparison fails to draw out any group as significantly different from each other, probably due to small sample sizes for some of the groups (Table L in S1 File).

## Discussion

### Amazonian baselines

Within tropical rainforests, plants that grow close to the ground, under a closed forest canopy, are expected to be heavily depleted in ^13^C due to low light and recycled CO_2_ [72, 74]. Previous research in the Amazon Basin has noted that the leaves from plants growing in the lower forest strata have δ^13^C values of -34.3‰ in laterite forest and -35.2‰ in podsol forests, while canopy leaves have an average of -28.7‰ in the laterite forest and -30.5‰ in the podsol forest [102]. This ‘canopy effect’ is expected to be reflected in the tissues of animals that feed in these environments [103]. However, recent studies of living fauna in the Peruvian Amazon carried out by Tejada and colleagues [79] have questioned the degree to which rainforest animals will reflect such negative sub-canopy values and display a median δ^13^C_diet_ value of -27.4 ‰ for mammalian herbivores. The average of δ^13^C_ap_ of herbivorous fauna of archaeological origin sampled by Roosevelt in Peru [40] (δ^13^C_ap_ –13.3 ‰ ± 1.4 ‰ in archaeological Ucayali- excluding capybaras, δ^13^C_ap_ –12.5 ‰ ± 2.2 ‰ in archaeological Ucayali–including capybaras) is even higher than the average values obtained by Tejada and colleagues for modern fauna [79] (δ^13^C_ap_ -15.6 ‰ ± 1.6 for the modern Peruvian study- excluding capybaras, δ^13^C_ap_ -15.0 ‰ ± 3.1 to the modern Peruvian Amazon–including capybaras. See Tables W and X in S1 File). The modern Peruvian values were already corrected back to 1750 by Tejada et al. [79] and it is those values we use here for comparison. Our values of herbivorous fauna (δ^13^C_ap_ -14.9 ‰ ± 0.8 ‰ in VGRX—disregarding capybaras; δ^13^C_ap_ -14.8 ‰ ± 0.9 ‰ in VGRX—including capybaras), is higher than the ones reported for the modern Peruvian samples (Kruskal-Wallis chi-squared = 16.425, df = 2, p-value < 0.05 including Capybara, Kruskal-Wallis chi-squared = 27.276, df = 2, p-value < 0.05 excluding Capybara) (Tables O and P in S1 File). Nevertheless, despite this variability, values still fall below the -14.0‰ threshold predicted for fauna living under a dense canopy. The only exception is the archaeological sample from Ucayali, average δ^13^C_ap_ that shows values below the -14.0‰ threshold. The data suggests that tropical forest fauna can still be easily distinguished from fauna living in more open areas or consuming C_4_ resources.

As noted above, stable carbon isotope values of δ^13^C_ap_ from Peruvian and VGRX mammalian herbivores were compared, both including capybara specimens and excluding the capybara specimens. This is due to the fact that, in the modern Peruvian sample, the only C_4_ consuming herbivore identified was the capybara (*Hydrochoerus hydrochaeris*) (*n* = 6, δ^13^C_ap_ -3.1 ‰ ± 3.4 ‰ corrected Suess Effect for 1750). The modern analyzed individuals have higher values than the single specimen of the same genus analysed from VGRX (δ^13^C_ap_ -13.7 ‰) and the two specimens analysed by Roosevelt [40] from Ucayali archaeological site in Peruvian (*n* = 2, δ^13^C_ap_ -8.6 ± 0.1 ‰, using bone apatite; *n* = 1, δ^13^C_co_ -15.9 ‰). Nevertheless, in both the modern and archaeological Peruvian studies, the values confirm that the water-land interface is used intensely by capybaras which is unsurprising given their known food preferences for semiaquatic grasses [104, 105]. Although the VGRX capybara has a lower δ^13^C value when compared to the values of capybara in the Peruvian Amazon, both for collagen (*n* = 2, δ^13^Cco -18.4 ‰ ± 0.7 ‰) and for bioapatite, these values are still the highest of the VGRX fauna measured here, confirming the preference of these animals for more open aquatic environments in VGRX too but, different from the Peruvian case, they seem to retain a more dominant reliance on C_3_ resources in VRGX. All other terrestrial herbivores measured in the modern and archaeological studies, including our own, show a clear preference for C_3_ consumption with a strong influence of the canopy effect.

In terms of δ^15^N in VGRX, one of the two agouti (*Dasyprocta* sp.) analyzed from VGRX (Table E in S1 File) yielded a higher value than expected for herbivorous taxa (11.2‰), suggesting the possible omnivorous potential of this species [106]. It is usually assumed that herbivores will have values of δ^15^N that reflect the local vegetation, and existing data shows that variations within the population of a given species can easily exceed 1‰ [107]. This variation was not only detected among the population of the same species in VRGX (Table E in S1 File), but it can be even greater when compared to species from other regions of the Amazon, such as the Ucayali Basin, for example (archaeological samples) [40]. The values of δ^15^N in VGRX are higher than in the Ucayali Basin for peccary (Tayassuidae, mean of 8.3 ± 0.5 ‰ for VGRX and 5.7 ± 0.1 ‰ for Ucayali Basin), for capybara (*Hydrochoerus hydrochaeris*, mean of 5.0 ± 0.2 ‰ for VGRX and 3.6 ‰ for Ucayali Basin) and for deer (Cervidae, mean of 8.6 ± 0.4 ‰ for VGRX and 5.7 ± 0.4 ‰ for Ucayali Basin). An ANOVA test showed there are δ^15^N differences between species from VGRX and Ucayali (F(8,24) = 8.469, *p*<0.05), although a post-Hoc Tukey Pairwise comparison failed to find significant differences (Tables R and S in S1 File). This work further highlights the importance of developing a robust baseline for human δ^15^N and δ^13^C interpretation, as variation can occur even within a given biome and within a given species.

Overall, our data show a clear reliance on C_3_ resources across the terrestrial fauna studied. Furthermore, a distinction in δ^15^N is noted between terrestrial herbivores and omnivorous and aquatic taxa. However, our data also highlight the potential confounding isotopic effects of complex freshwater systems [108]. This is particularly difficult in the Amazon where some fish are noted for their consumption of fruits and seeds of tree species [109, 110]. The range of fish δ^13^C_co_ (-23.8 to -18.5 ‰) and δ^13^C_ap_ (-13.1 to -9.5 ‰) covers a major portion of expected C_3_ and mixed C_3_/C_4_ space, making it difficult to distinguish reliance on terrestrial resources versus aquatic resources. Indeed, the average values of δ^13^C_co_ of fish measured here are not significantly different from the average values of δ^13^C_co_ of terrestrial herbivores (*p* >0.05), terrestrial omnivores (*p* >0.05) and alligators (*p* >0.05) (Table I in S1 File), confirming the predilection of at least some of these fish for the consumption of fruits and seeds of C_3_ species [109, 110]. However, it is clear that terrestrial herbivores and omnivores experiencing the canopy effect broadly show lower δ^13^C than most aquatic samples, particularly for δ^13^C_ap_. Similarly, while it is difficult to distinguish terrestrial omnivores from aquatic resources based on δ^15^N, herbivores emerge as being distinct from both of these groups.

### Diets on the Xingu River between 390 cal. BC and 1,675 cal. years AD

Despite the fact that freshwater δ^15^N and δ^13^C cover a large range of isotopic space, we are still able to observe some notable distinctions in the human isotopic data. The values of stable isotopes of human δ^13^C_co_ from the four VGRX individuals with preserved collagen are higher (average of -16.5 ± 1.1 ‰) than the values obtained for terrestrial herbivores (-21.7 ± 1.4 ‰), carnivores (-20.3 ± 0.1 ‰), omnivores (-21.8 ± 1.3‰) and fish (-21.2 ± 1.8 ‰), suggesting the addition of an additional food source into their diets compared to local fauna. When comparing the human values of δ^13^C_ap_ to those of the faunal baseline, a large variation in individual human values is visible. However, the human dataset is also higher (-10.4 ± 1.3 ‰) than the δ^13^C_ap_ of herbivorous fauna (-14.8 ± 0.9 ‰), but similar to carnivorous fish (-12.2 ± 1.3 ‰) and omnivorous fish (-10.1 ± 0.9 ‰). The human δ^15^N collagen values are also higher (12.1 ± 0.9 ‰) than those of fauna (8.2 ± 1.6 ‰ for herbivorous terrestrial fauna, 10.1 ± 0.3 ‰ for carnivorous terrestrial fauna, 10.6 ± 2.1 ‰ for omnivorous terrestrial fauna and 9.8 ± 1.0 ‰ for fish) (Fig 4). Overall, then, there seems to be a clear spectrum of variation in human diets ranging from a reliance on closed canopy terrestrial plants, animals and different types of aquatic resources to a clear input of δ^13^C resources that are not documented in the faunal baseline and are likely representative of additional C_4_ resource use such as maize (e.g. for individuals BEL-001, PAM-002, PED-002 and VIL-002).

When considering possible differences in economies between *Terra Firme* and *Várzea* sites [3], it does not appear that the economies of the groups that inhabited the Vila Rica 2 site (the only *terra firma* context ultimately available due to the lack of preservation of organic material in the other excavated sites) differ isotopically from the groups who inhabited the riverside (average of -9.5 ‰ ± 1.0 ‰, *n* = 2 to Vila Rica 2, average of -9.9 ‰ ± 1.0 ‰, *n* = 3 to Pedra do Navio, for example, all from the last millennium). The characteristics of the material culture of both are similar and the isotopic values would suggest limited differences in economic strategy, although it should be noted that low sample sizes currently hinder a statistically-valid comparison between the two types of sites and more research testing this suggestion is needed. Furthermore, we have seen that terrestrial fauna and freshwater resources can be difficult to distinguish from each other, meaning that more subtle economic variations may be hard to discern isotopically. The same can be said for C_3_ wild and domesticated (e.g. manioc) plant resources, which will have similar isotopic values and thus be effectively indistinguishable. With regards to isotopic differences between different cultural contexts among the VGRX sites some intragroup variation is clear. São José 1 is the only site with Arauquinoid ceramic-styles. The lowest human δ^13^C_ap_ value (-13.3 ‰) comes from this site, perhaps suggesting greatest reliance on local terrestrial fauna from forest biomes (mean of δ^13^C_ap_ herbivores of -14.9 ‰ and omnivorous of -14.0 ‰). Nevertheless, within the same site, variation of δ^13^C_ap_ could be up to 2.7 ‰ in São José 1 and 2.6 ‰ in Palmeiras. Low variation in δ^18^O values among all individuals does not support long distance migration being a significant phenomenon in these sites and is rather consistent with resident intragroup dietary differences, although human δ^18^O is notoriously difficult to interpret in isolation.

Differences in δ^13^C_ap_ values between individuals at a given site also cannot be explained in relation to the treatment given to the dead (as a possible way of assessing social stratification or differentiated social positions). The four individuals with higher δ^13^C_ap_ values from VGRX come from four different archaeological sites (Bela Vista, Palmeiras 1, Vila Rica 2, and Pedra do Navio) and were deposited in four different forms of burial. The individual with higher δ^13^C_ap_ values (-8.4 ‰) was a probably child and was buried inside of an urn (Bela Vista site). The individual from Burial 2/Palmeiras (δ^13^C_ap_ -8.7 ‰) was an adult man and was found as a primary burial, without visible goods around the body. The individual from Structure 29/Vila Rica 2 (δ^13^C_ap_ -8.7 ‰), buried with a vessel overturned over the head, could not be sexed or aged due to poor preservation. The individual from Burial 1/Pedra do Navio (δ^13^C_ap_—8.8 ‰) was an adult and was buried in primary form, with at least three items of pottery as grave goods. The three individuals with lower δ^13^C_ap_ values come from three archaeological sites and were in two different burial forms: an adult, primary burial without visible goods (Burial 3/São José 1 site, δ^13^C_ap_− 13.3 ‰), a child buried with vessel overturned on the head (Structure 2/Santo Antônio 1 site, δ^13^C_ap_− 12.1 ‰), and an adult, man, primary burial without visible goods (Burial 2/Palhal 2 site, δ^13^C_ap_− 11.9 ‰) (Fig A in S1 File). When looking for differentiation between the sexes, there are few individuals that can be confidently identified as male or female (*n* = 7). However, it should be noticed that the three females have δ^13^C_ap_ values closer to each other (-10.6‰, -10.0‰ and -10.6‰, from Pedra do Navio, Pimental 2 and São José 1 archaeological site respectively), while males have more dispersed values, from the lowest (-11.9‰ from Palhal 2) to the highest (-8.6‰, from Palmeiras 1) (Fig B in S1 File), suggesting that there could be some possible dietary variation. Unfortunately, the number of individuals whose sex has not been identified is quite high (*n* = 10), meaning there may be bias in the sample size, as well as the fact that comparisons are being performed between different sites and different biomes. To confidently identify sex-based differences in diet and economy a more detailed investigation of sexed skeletons at a given site and context would be required.

### Comparison with other Amazon datasets

Ethnographic and ethno-historical reports from the time of European invasion document the existence of dense populations living in a complex system of social organization along the Amazon River [111, 112]. Several archaeological sites have already been excavated in this region confirming, alongside palaeoecological work, contemporary occupation and cultural and social relationships between the *Terra firme* and *Várzea* occupations [113], the production of ADE, and the cultivation of squash (*Cucurbita* sp.), maize (*Zea mays*) and cassava (*Manihot esculenta*) [9, 113]. For the time being, there are no human stable isotopic studies of individuals exhumed from these sites (of the so-called Tapajós culture) to compare with the ethno-historical data and determine the intensity of maize consumption and its relationship with the social structure of these groups. However, previous studies carried out on individuals from the Corazal phase in Venezuela (Orinoco Basin), suggested a change in the dietary patterns of populations between 800 years BC and 400 years AD, with the increasing incorporation of maize as a staple food (Table H in S1 File). This change has been linked to the development of larger settlements to support denser populations [22]. A similar transition in dietary patterns was also noticed in the western region of the Amazon, in Peru (Ucayali Basin) [40].

Nevertheless, while maize appears to be well-established in the diets of populations of the north and west of the Amazon by the first millennium AD [22, 40], in other regions its dietary significance appears to be more subtle. At the mouth of the Amazon river, isotopic analysis of human remains from two important cultures (Maracá and Marajó) yielded δ^13^C_co_ values which suggest that maize, despite being found in the archaeological sediments in the lower Amazon river dating to around 4.300 cal BP [8, 9], was not a significant foodstuff for Maracá and Marajó populations, who probably focused on the exploitation of forest and riverine resources [24]. At the mouth of the Amazon River, δ^13^C_co_ analysis of human remains from Marajó sites are slightly higher than the values obtained for the Maracá, though the data has again been interpreted as being indicative of a mixed diet, with variability being related to differential access to certain food groups [40]. In both cases, the available faunal and freshwater baselines are currently limited, making more detailed discussions of dietary variability challenging. Meanwhile, one the eastern edge of the Amazon, the values of δ^13^C_co_ found in individuals from different archaeological cultures from São Luis Island also suggest the superficial consumption of maize, along with other C_3_ items and mammal hunting, reinforcing the secondary role of this crop for the populations that lived in the eastern Amazon in the Late Holocene [41].

**Fig 7:**
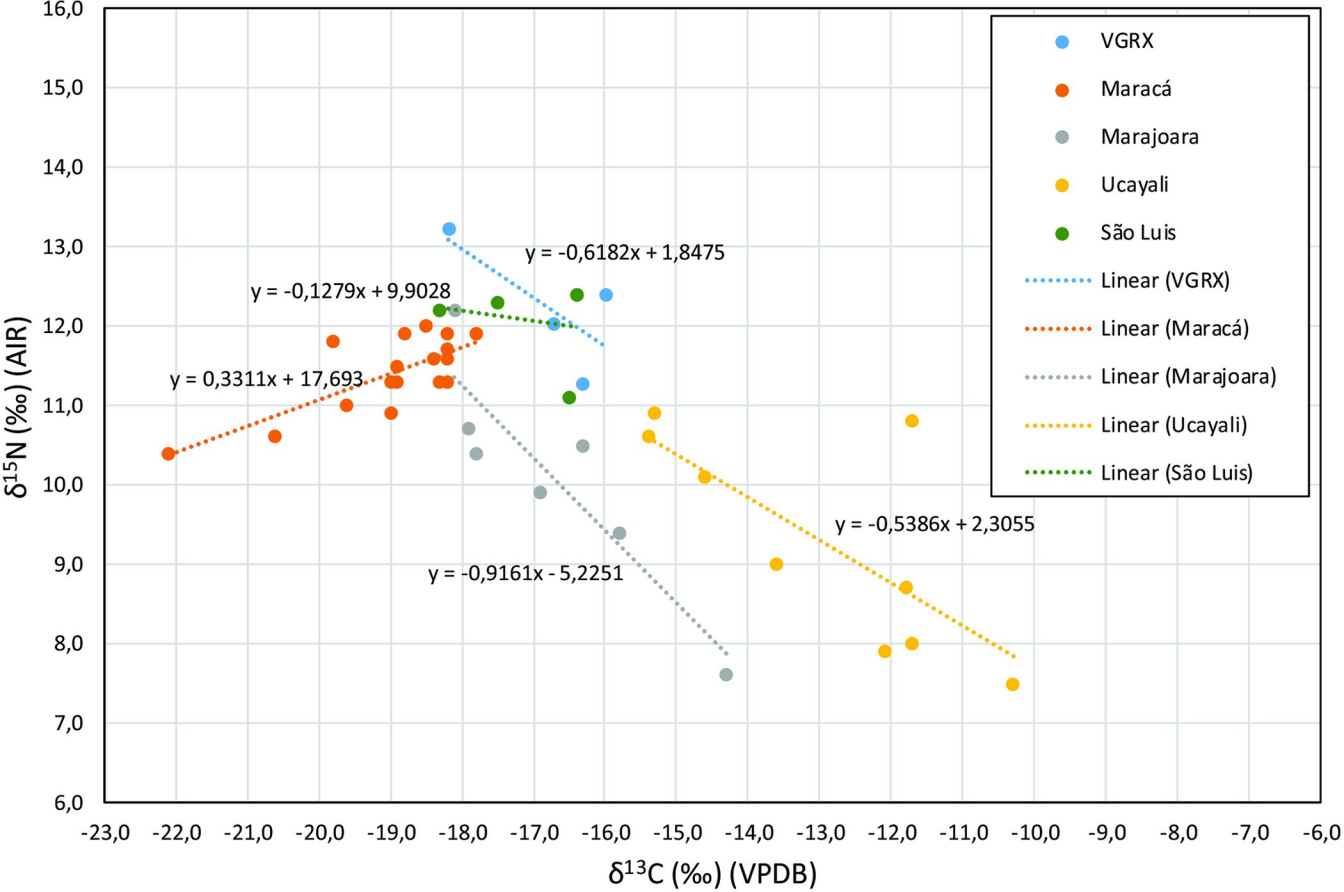
δ^13^C_co_ and δ^15^N values of archaeological human bones from VGRX, Marajó, Maracá, São Luis and Ucayali.

When the δ^13^C_co_ values from the VGRX samples are compared with those available from the Orinoco basin, it appears that the four individuals from VGRX present δ^13^C_co_ values intermediate to the oldest and most recent phases, suggesting a mixed diet that includes both C_3_ and C_4_ resources. However, VGRX values (average of -16.5 ± 1.1) are lower than values found among the Ucayali individuals (-14.7 ± 0.8 to Early Ucayali, -11.5 ± 0.7 to Late Ucayali), suggesting less reliance on maize. Indeed, comparing the values of δ^13^C_co_ of VGRX with the populations from the mouth of the Amazon and São Luis, it is clear that they are very close to each other (average of -19.0 ± 1.1 to Maracá, -16.7 ± 1.4 to Marajó, -17.0 ± 0.9 to São Luis). A Kruskal-Wallis test showed there are δ^13^C_co_ differences between them (chi-squared = 39.954, df = 7, p-value < 0.05) and Pairwise comparisons using Wilcoxon showed that Maracá is different from Marajó (p = 0.009) and Late Ucayali (p = 0.026). However, the small sample size means further pairwise comparison differences could not be observed (Table T in S1 File). When comparing δ^15^N between the published studies, our VGRX data have the highest values (average of 12.1 ± 0.9), very close to those of the São Luis value (12.1 ± 0.6). Again, a Kruskal-Wallis test showed the existence of differences between human groups (chi-squared = 25.358, df = 5, p-value < 0.05) though pairwise comparisons using Wilcoxon tests showed only Maracá (average of 11.4 ± 0.5) to be significantly different from Late Ucayali (average of 8.6 ± 1.3) (p = 0.025) (Table U in S1 File). Interestingly, a linear regression analysis shows a strong negative correlation between δ^13^C_co_ and δ^15^N for VGRX, as at Ucayali and Marajó, which implies that higher δ^13^C_co_ is related to lower trophic foods, perhaps in the form of maize (Fig 7).

Overall, then, the human and faunal data from VGRX, as well as its comparison with data from elsewhere in the Amazon Basin, indicate the varying incorporation of maize into individual diets even within a given region. This agrees with the descriptions of travellers and researchers from the 16^th^ to the 19^th^ centuries, who note the widespread cultivation of maize by Indigenous populations [51, 65, 67, 111, 112]. Nevertheless, maize was not the primary basis of diets and economies at VGRX and, as at Amazon mouth, a combination of hunting, fishing, vegetable harvesting and horticulture provided a rich diet and a more regular supply of food, ensuring that these groups were not dependent on a single foodstuff, therefore, decreasing the risk of exposure of communities to problems obtaining any particular given food source. The data presented is also consistent with an emerging consensus that there was no single adaptive pattern for pre-colonial Amazonian populations and proposes that diversified economic strategies, based on the management of wild and cultivated plants combined with the exploitation of aquatic and terrestrial fauna resources, could have developed over centuries and sustained long-term successful, often-large human populations [114]. Archaeological insights into a diversity of landscape management strategies, including the construction of raised fields, dikes, canals, wells, ponds, sidewalks, roads and hills for housing and burial [10, 21, 23, 35, 115, 116] support this, with more isotopic studies required to gain further insights into cultural, social, and ecological distinctions in diet and economies across this vast area.

## Conclusions

There is growing archaeological evidence that the long-term history of occupation of Amazonia culminated in large population aggregates by 1,000 AD [117], as suggested by the clear increase in the size and number of archaeological sites as well as the widespread discovery of Anthropogenic Dark Earth sites. This fluorescence is sometimes associated with the arrival of maize into subsistence systems across the floodplain societies of the Greater Amazon; and previous analyses of dental pathologies, associated with microbotanical data suggest that grain crops may have become quite important between 500 AD and European conquest [23]. Isotopic analyses of human remains can provide privileged direct insights into the incorporation of maize into Amazonian diets and, in the northern and western Amazon, a correlation has been documented between population increase and maize consumption. Nevertheless, our data for VGRX, as well as other studies in Marajo and Maracá [24], and on São Luis island [41], indicate a far more limited consumption of maize, despite it being present in these regions as early as 4,300 cal. BP. Overall, the findings point to significant variability in the diets of Amazonian populations, something perhaps unsurprising given the vast cultural, ecological, and geographical variation of the Amazon Basin, an area roughly the size of Europe. In particular, management of wild trees, cultivation of tubers, the hunting and the capture of wild animals, and even corralling, of freshwater resources seem to have provided varied approaches to the maintenance of significant populations and settlement networks at the time of European invasion, calling into question the necessity of conventional ideas of ‘agriculture’ to sustain such phenomena. Further multidisciplinary research in different contexts is now essential to determine the degree to which pre-contact Amazonian diets varied across cultural and social contexts, as well as between ecological settings. In this way, understandings of economic resilience and land-use variability across different portions of the Amazon Basin can be better developed and factored into the ongoing debate about the impacts of pre-colonial societies on the tropical forests of this increasingly threatened biodiversity hotspot [118].

## Supporting information

S1 File Description of the archaeological sites, details of the results of the ^14^C analyses, brief description of fauna behaviour, tables with the samples listed and the results of the stable isotope analyses, tables with full statistic results, and complementary graphs. (DOCX)

S2 File (DOCX)
