# Supplementary material for: Late Holocene dietary and cultural variability on the Xingu River, Amazon Basin: A stable isotopic approach
Source: PLoS One. 2022 Aug 3;17(8):e0271545. doi: 10.1371/journal.pone.0271545 (PMC9348659; doi:10.1371/journal.pone.0271545)
Supplement: S1 File — (DOCX) [file pone.0271545.s001.docx]

**S1 File**

Supplementary Information (SI) for:

**Dietary and cultural variability on the Xingu River, Amazon Basin: a stable isotopic approach**

Letícia Morgana Müller^1,2,3*^, Renato Kipnis^3^, Mariane Pereira Ferreira^3,4^, Sara Marzo^5^, Bianca Fiedler^1^, Mary Lucas^1^, Jana Ilgner^1^, Hilton P. Silva^2^, Patrick Roberts^1,6*^.

^1^ Department of Archaeology, Max Planck Institute for the Science of Human History, Jena, Germany.

^2^ Graduate Program in Anthropology, Federal University of Pará, Belém, Pará, Brazil

^3^ Archaeology Division, Scientia Consultoria Científica, São Paulo, São Paulo - SP, Brazil

^4^ Graduate Program in Archeology. Museum of Archaeology and Etnology, University of São Paulo, São Paulo, Brazil.

^5^ The Roslin Institute & Royal (Dick) School of Veterinary Studies, University of Edinburgh, Midlothian, Edinburgh, UK.

^6^ School of Social Science, The University of Queensland, Brisbane, QLD, Australia

* Corresponding author

E-mail: [roberts@shh.mpg.de](mailto:roberts@shh.mpg.de) (PR)

E-mail: [muller@shh.mpg.de](mailto:muller@shh.mpg.de) (LMM)

**This PDF file includes:**

Supplementary information about the archaeological sites

Supplementary information about carbon dating

Supplementary Figs. A-C

Supplementary Tables A-V

SI References

## Supplementary Text

## Sampled sites and brief description of burials

Most of the material used in this research was obtained as part of the archaeological investigations associated with the environmental license issued for the construction of the Belo Monte Hydroelectric Power Plant in Northeast Amazonia, Brazil (Palmeiras, Palhal 2, Vila Rica 2, São José 1, Gaioso 13, Santo Antônio 1, Santa Luzia 1 sites, Pedra do Navio and Pimental 2). The impacted sites were excavated between the years 2011 and 2016. Only the Bela Vista site is located in the Volta Grande do Rio Xingu (VGRX) area and it was researched in the 2000s by the team from the Museu Paraense Emílio Goeldi (MPEG). The Carrazedo site is the only site located at the mouth of the Xingu River and research there was carried out in 2014 by the MPEG (Project “Origens, Cultura e Ambiente” (OCA Gurupá)) (1).

The Palmeiras archaeological site is located on the western margins upstream of the VGRX (UTM SAD69 22M 352550/9617460). The site occupies an area of approximately 256,000 m² and has a layer of Anthropogenic Dark Earth (ADE) up to 60 cm thick from the surface. The ADE is dispersed in the form of circles distributed in a ring pattern. Archaeological material presents higher densities associated with the ADEs, and in between ADE circles the latosol is associated with a decrease in archaeological material. Site stratigraphy and archaeological materials indicates more than one temporal occupation at the site, associated with ceramic producers. The ceramic collection presents a rich variety in terms of pottery morphology and painted decoration, with characteristic attributes that relate to Koriabo and Tupiguarani ceramics. Radiocarbon analysis carried out on a charred wood sample indicates that the site was occupied in 810±30 BP (1169-1270 cal AD, 95.4%). Excavations carried out at the site revealed three human burials. Two were exhumed, and were primary burials of adult male individuals, buried in flexed and right lateral decubitus position, without funerary goods. The three burials were found within the ADE strata (40-50 cm deep from the surface) (2).

The Pedra do Navio archaeological site is located on the western margins of the Xingu River, upstream of the VGRX (UTM SAD69 22M 364170/9637490). The site occupies an area of approximately 194,844 m² and the material culture is spread over two different areas, one on the hillside and the other on the Xingu River floodplain. The ADE is up to 60 cm thick from the surface. The ADE is dispersed only along the floodplain and archaeological material presents higher densities associated with ADEs. Site stratigraphy and archaeological material indicates occupation associated with horticulturalists and ceramic producers. It is possible to identify at least six well-defined styles in the ceramic record, namely: Arauquinoid, Saladoid, Koriabo, Tupiguarani, Hachurado-zonado and Upper-Xingu. In the funerary context, on the other hand, it is only possible to identify vessels related to the Tupiguarani and Koriabo types. Radiocarbon analysis carried out on charred wood samples indicate that the site was occupied between 610 and 630 BP (1287-1404 cal.AD (95.4%). Excavations carried out at the site revealed two human primary burials of adults, one with sex not identified and one identified as female, both buried in flexed positions with funerary goods of Tupiguarani and Koriabo pottery (Burial one and two, 60-70cm and 90-100 cm deep from the surface, respectively). A further burial was identified inside a funerary urn, accompanied by three pots of Koriabo and Tupiguarani style and a polished axe blade (urn 2, located in 80-90 cm deep from de surface). Three burials were found in funerary urns without clear accompaniments (urn 1, 3 and 4, 60-70 cm). All the burials were found below the ADE strata (3,4).

The Santa Luzia 1 archaeological site is located on the western margins of the Xingu River upstream of the VGRX (UTM SAD69 22M 398380/9623270). The site occupies an area of approximately 79.000 m² and the material culture is spread over two different areas, both on river terrace of Xingu River. ADEs were found in patches in a number of areas, with a thickness not exceeding 50 cm from the surface. Site stratigraphy and archaeological material indicates occupation associated with horticulturalists and ceramic producers. No funerary contexts were found. In the ADE layer, a large amount of fauna was found, some of which was used in this study. Radiocarbon analysis carried out on charred wood samples indicate that the site was occupied in 1329±27 BP (650-716 cal.AD, 79.9%; 743-766 cal.AD, 15.6%) (3).

The Gaioso 13 archaeological site is located on the western bank of the Xingu River, upstream of the VGRX (UTM SAD69 22M 394680/9627270). The site occupies an area of approximately 53,200 m² and the material culture is spread from the river bank up to the slope away from the Xingu River. The ADE is up to 30 cm thick from the surface. The ADE layer is confined to some specific points of the site. Site stratigraphy and archaeological material indicates occupation associated with Koriabo ceramic producers. Only one burial was found, in an urn found in 60-70cm deep from the surface, without other goods (5).

The Santo Antônio 1 archaeological site is located on the western margins of the Xingu River, upstream of VGRX (UTM SAD69 22M 411210/9656070). The site occupies an area of approximately 67,450 m² and the material culture is spread across the top of a high elevation slope which lies besides the Xingu River. No ADEs were found. Site stratigraphy and archaeological material indicates occupation associated with ceramic producers. It is possible to identify at least three well-defined styles in the ceramic record, namely: Arauquinoid, Koriabo and Tupiguarani. Excavations carried out at the site revealed one human primary burial, child (5 +-1,5 years old) with a vessel on the head, in 20-30cm deep from the durface. No bone was found in the burial, only some teeth (5).

The Vila Rica 2 archaeological site is located on the western margins of the VGRX (UTM SAD69 22M 403180/9636690). The site occupies an area of approximately 40,000 m² and the material culture is spread across a plateau of *Terra Firme* near to the Paquiçamba stream, c. 12km from Xingu River. The ADE is up to 30 cm thick from the surface. The ADE is dispersed in some areas of the site. Site stratigraphy and archaeological material indicates occupation associated with ceramic producers. It is possible to identify at least two well-defined styles in the ceramic record, Koriabo and Tupiguarani, as well in the funerary context. Radiocarbon analysis carried out on charred wood samples indicate that the site was occupied in 850±30 BP (1052-1080 cal.AD, 5.2%; 1152-1260calAD, 90.2%). Excavations carried out at the site revealed two human primary burials with pots located over the head as a funerary good (structure 28 and 29). No long bones were found, only some cranial bones and teeth. All of the burials were found below the ADE strata (6,7).

The Pimental 2 archaeological site is located on the western margins of the VGRX (UTM SAD69 22M 399280/9621850). The site occupies an area of approximately 81,600 m² and the material culture is spread along a river terrace associated with a small flooded area beside the Xingu River. The ADE is up to 80 cm thick from the surface and was located all over the site. Site stratigraphy and archaeological material indicates occupation associated with ceramic producers. It is possible to identify at least two well-defined styles in the ceramic record, Koriabo and Tupiguarani. At least five funerary structures and two urns were found between 20 and 70cm deep from the surface. The structures (burial 1 to 5) are characterized as primary, already quite deteriorated, with bone powder solely remaining in most cases. Two of these burials were possible to identify as adult women. There were no funerary goods. The burials in urns (Urn 1 and 2), one of them infantile, were also very degraded and without burial goods. Radiocarbon analysis carried out on charred wood samples indicate that the site was occupied between 680±30 BP and 248±26 BP (1324-1800 cal.AD, 95.4%). All of the burials were found in and below the ADE strata (8).

The São José 1 archaeological site is located on the western margins upstream of the VGRX (UTM SAD69 22M 385340/9639040). The site occupies an area of approximately 140,800 m² and the material culture is spread over a plateau, hillside and floodplain adjacent to the Xingu River. The site has an ADE patch and a *Terra Mulata* patch, both on the banks of the river but in a non-floodable area. The ADE is 30/ 60 cm thick from the surface. Site stratigraphy and archaeological material indicates occupation associated with ceramic producers. It is possible to identify at least two well-defined styles in the ceramic record, namely: Arauquinoid and Koriabo. Radiocarbon analysis carried out on charred wood samples from the beginning of ADE layers (40-50 cm deep from the surface) indicates that the site probably was occupied since 2.240±30 BP (390-345 cal.BC, 25%; 323-205cal.BC; 70.4%). Excavations carried out at the site revealed four primary burials of adults associated with Arauquinoid pottery, two with the sex not identified (burial 1 and 3), one probable female (burial 4), and one probable male (burial 2). All of the burials were in a deep layer (burial 1 in 120-130 cm deep from the surface, burial 2 in 140-150 cm deep, burial 3 in 50-70 cm deep and burial 4 in 1150-170 cm deep) below the ADE and were not associated with burial goods. The burials 2 and 4 were deposited together inside the grave (8,9).

The Palhal 2 archaeological site is located on the eastern margins upstream of the VGRX (UTM SAD69 22M 385220/9628390). The site occupies an area of approximately 105,000 m² on a river-adjacent hillside and floodplain. The layer of ADE is up to 80 cm thick from the surface. Site stratigraphy and archaeological material indicates at least two different occupations, one older associated with hunter-gatherers and the other occupation associated with ceramic producers. The ceramic collection presents a rich variety in terms of morphology and decoration, with characteristic attributes that relate to Koriabo and Tupiguarani ceramics. Radiocarbon analysis carried out on charred wood sample indicate that the site was occupied by ceramists between 967±28 and 350±30 BP (1324-1345 cal AD, 10.5%; 1393-1443 cal AD, 84.9%) and by hunter-gatherers between 9470±30 and 5510±30 BP (8838-4274 cal BC, 95.4%). Excavations carried out at the site revealed two human burials associated with the later period. Both were exhumed, and were primary burials of adult male individuals, buried in flexed and right lateral decubitus position, 30-60cm deep from the surface, without funerary goods. They were found within the ADE strata (8,2,10).

The Bela Vista site (PA-AL-26) is located at VGRX on a river terrace. In 2000, it was visited by MPEG technicians as part of preliminary studies in advance of the construction of the Belo Monte Hydroelectric Plant when two funerary urns were rescued. One of them was excavated in 2018, exposing a child burial without accompaniments (11,12).

The Carrazedo site was excavated by the MPEG team in 2014 as part of the OCA project. The site is located at the confluence of the Xingu River and the Amazon River, and spans an estimated area of 300,000 m². It is located on top of an elevated river terrace on the eastern bank of the Xingu River, close to its mouth at the Amazon River. It is considered to be a pre-colonial and historic settlement, with ruins of structures built after conquest visible on the edge of the site area. It has an ADE layer of approximately 50 cm. Two dates obtained with charcoal analyses were published, placing the pre-conquest occupation at 1,260 to 1,460 cal AD and 750 and 870 cal AD (13). The ceramic collection from the pre-colonial occupation presents some characteristic attributes that relate to the Koriabo ceramic style. At this site, a burial in an urn was identified.

**Supplementary information about fauna behaviour in the Amazon**

For the classification of the feeding behavior of the fauna sampled in this research, the research by Robinson & Redford (14) was used, complemented with more recent and specific research for each species:

*Alouatta*: The genus *Alouatta*, whose species are popularly known as howler monkey or barbado, has a wide geographic distribution in the Neotropical region. The diet of *Alouatta* is classified as folivorous-frugivorous, but they also feed on flowers, stems, bark and lichens (15,14).

*Alligatoridae:* Representatives of this group have a carnivorous diet, and studies show that the diet can vary with animal size. Young individuals feed on a wide variety of prey, varying in type as they grow. These prey can be fish, crabs, birds, reptiles and mammals (16).

*Chelonoidis*: They are widely distributed in the Amazon. Studies of the natural diets of turtles have reported a significant proportion of fruit throughout the year, which may indicate that they are important disseminators of seeds in the environment (17). They also described other components of the diet, such as foliage, stems, fungi, pebbles, insects and animal matter throughout the year. Several studies have reported opportunistic scavenging behavior, recovering samples of agouti (*Dasyprocta leporina*) and peccary (*Tayassu pecari*) bones from the feces of wild individuals. Consequently, these animals are characterized as a diurnal, terrestrial and opportunistic omnivorous species, with coprophagy behaviour (18).

*Cuniculus paca:* The paca is a large frugivorous rodent that occurs throughout the Neotropics. They are an important prey of large carnivores, and act as key seed dispersers. They feed mainly on fruits and seeds and occasionally consume leaves and flowers (19).

*Dasyprocta:* Agoutis are mainly frugivorous-granivores (14), with their main foods being leaves, fungi, seeds and fruit pulp. They also eat invertebrates and animal matter, especially when in captivity (20,21).

*Dasypus novemcinctus:* The 21 species of armadillos can be divided into four groups according to their food specializations, with *Dasypus* being considered a generalist (terrestrial) insectivore. (22). The food preference of armadillos is for termites and ants, but they can also feed on some other arthropods, or even fruits, tubers and small vertebrates, depending on their availability (23).

*Hydrochoerus hydrochaeris:* The capybara is a large rodent whose occurrence comprises South America and almost all Brazilian states. It is a species of herbivorous habit that feeds on aquatic and undergrowth vegetation (24,14).

*Hydrolycus scomberoides:* A piscivorous fish species capable of eating whole prey due to its large mouth with suspended jaw, but can also capture prey using its long canine teeth. In their diet are fish, shrimp, fruits, and seeds (25).

Mazama sp.: Deer are the most widely distributed wild ruminants in the world, present on all continents with the exception of Antarctica (26). Their diet is based on fruits and herbs (14).

*Pecari Tajaçu*: Collared peccaries can be considered omnivores due to the wide variety of foods consumed, but they are predominantly frugivorous in tropical forests (27). Collared peccaries live in a great diversity of habitats, from humid tropical forest regions to semi-arid regions. The ability of this species to survive in different conditions is due to its ability to consume a long list of food items such as fruits, leaves, roots, cactus and tubers (28). In this research, we assume Collared peccaries to be herbivores, following the research carried out by Tejada et.al (29) with modern fauna in Peru.

*Podocnemis:* Six species of *Podocnemis* are found in the Amazon region, with distinct distributions and habitat preferences. *Podocnemis* are opportunistic, generalist and omnivorous, although, overall, they tend towards herbivory. The proportion of plant and animal matter consumed varies considerably between populations, periods and species (30).

*Priodontes maximus:* First described as nocturnal, solitary and mainly insectivorous (mainly termites), the discovery of large amounts of fruit seeds in the stomach contents of this species in different biomes suggests the consumption, at least seasonally, of other food sources (31).

*Tapirus terrestres:* The tapir is the largest Neotropical land mammal. Its geographical range encompasses a variety of biomes, including tropical rainforest, semideciduous forest, and savanna. Tapirs are considered generalist herbivores and the differences in the proportions of dietary items are often attributed to differences in the habitats where individuals live. It has a diet composed of vegetable items, with fruits making up approximately 33% of the diet (14,32).

*Tayassu Pecari*: White-lipped peccaries are predominantly frugivorous (14,33) (above 50% of the diet is composed of fruits), feeding on fruits, seeds, roots, insect larvae and earthworms. White-lipped peccaries utilize numerous types of habitats throughout their wide range. They typically prefer dense, humid tropical forests, usually primary, although they also inhabit dry regions, such as savannas, but always close to a source of water (34). In this research we assume White-lipped peccary to be herbivores, following the work of Tejada et.al (29) with modern fauna in Peru.

**Supplementary information about radiocarbon dating**

The dates obtained by ^14^C were performed in the laboratories of Beta Analytic Inc and the Research Laboratory for Archaeology and History of Art (RLAHA), University of Oxford (Table S1). In both cases, small fragments of wood and carbonized seeds recovered from archaeological contexts of the researched sites were analysed. In addition, samples of human bone were sent to the RLAHA laboratory for dating.

All analyses at Beta Analytic were performed using the Conventional Method. The samples were given a standard acid-base-acid pretreatment. Measurement was performed using 4 in-house NEC accelerator mass spectrometers and 4 Thermo IRMSs. The "Conventional Radiocarbon Age" was calculated using the Libby half-life (5568 years), and is corrected for total isotopic fraction. The Age is rounded to the nearest 10 years and is reported as radiocarbon years before present (BP) (“present" = AD 1950). The Conventional Radiocarbon Ages have all been corrected for total fractionation effects and the Reported results are accredited to ISO/IEC 17025:2005 Testing Accreditation PJLA #59423 standards Conventional Radiocarbon. When counting statistics produce sigmas lower than +/- 30 years, a conservative +/- 30BP is cited for the result. The reported δ13C values were measured separately in an IRMS (isotope ratio mass spectrometer). Results greater than the modern reference are reported as percent modern carbon (pMC). The modern reference standard was 95% of the ^14^C signature of NIST SRM-4990C (oxalic acid). Quoted errors are 1 sigma counting statistics. δ13C values are on the material itself and are measured relative to VPDB-1 (data provided by the laboratory).

As noted above, the dates performed on human bone samples were undertaken at RLAHA, University of Oxford using the AMS method. The method used in chemical pretreatment, target preparation and AMS measurement is described in Bronk Ramsey et al., 2004, *Radiocarbon* 46 (1) 17-24, and Brock et al., 2010, *Radiocarbon* 52 (1): 103-112 (data provided by the laboratory). Two dates performed on charcoal were also undertaken at RLAHA, University of Oxford using the AMS method (samples OxA-33027 from Palhal 2 site and OxA-33028 from Pimental 2 site) (Table S1). The method used in chemical pretreatment, target preparation and AMS measurement is described in *Radiocarbon* 46 (1) 17-24, 46 (1): 155-63, and *Archaeometry* 44 (3 Supplement 1): 1-149.

For all resulting measurements, calibration of the dates was carried out using the online Oxcal 4.4.4 (35). application and the IntCal13 atmospheric curve (36). The calibrated values are presented in the Table S1 at 95.4% probability.

**Supplementary Tables**

**Table A in S1:** Detailed information relating to the samples analyzed for radiocarbon dating in this study

| **Site** | **Material** | **Lab Code** | **Radiocarbon age BP** | **δ^13^C** | **Pretreatment** | **Analysis Method** | **Cal 2α** |
| --- | --- | --- | --- | --- | --- | --- | --- |
| Palhal 2 | Human Bone | OxA-X-3050-26 | 424±25 BP | -16.8‰ | acid/base/acid | AMS | 1429 (92.8%) 1493calAD 1602 (2.6%) 1611calAD |
|  | Human Bone | OxA-39692 | 390±19 BP | -17.4‰ | acid/base/acid | AMS | 1445 (81.5%) 1515calAD 1598 (13.9%) 1618calAD |
|  | Fish bone | P-48109 (Oxford) | Failed due to no yield | - | - | - |  |
|  | Charcoal | Beta-542851 | 520±30BP | -28.1‰ | acid/alkali/acid | Radiometric PLUS | 1324 (10.5%) 1345calAD 1393 (84.9%) 1443calAD |
|  | Charcoal | OxA-33027 | 967±28 BP | -31.0‰ | acid/base/acid | AMS | 1018 (95.4%) 1155calAD |
|  | Charcoal | Beta-542854 | 370±30BP | -23.3‰ | acid/alkali/acid | Radiometric PLUS | 1485 (95.4%) 1650calAD |
|  | Charred seed | Beta-552222 | 350±30BP | -23.6‰ | acid/alkali/acid | Radiometric PLUS | 1458 (41.4%) 1531calAD 1538 (54.1%) 1635calAD |
| Pimental 2 | Charcoal | OxA-33028 | 248±26 BP | -26.9‰ | acid/base/acid | AMS | 1525 (7.5%) 1558calAD  1631 (62.5%) 1675calAD 1777 (22.3%) 1800calAD  1941(3.1%) until now. |
|  | Charcoal | Beta-554247 | 680±30BP | -26.0‰ | acid/alkali/acid | Radiometric PLUS | 1324 (10.5%) 1345calAD 1393 (84.9%) 1443calAD |
| Palmeiras | Human Bone | OxA-X-3050-27 | 371±26 BP | -15.6‰ | acid/base/acid | AMS | 1270 (60.4%) 1316calAD 1355 (35.0%) 1390calAD |
|  | Human Bone | OxA-X-3050-28 | 342±26 BP | -15.8‰ | acid/base/acid | AMS | 1470 (95.4%) 1637calAD |
|  | Charcoal | Beta-552227 | 810±30BP | -24.1‰ | acid/alkali/acid | Radiometric PLUS | 1169 (95.4%) 1270calAD |
| São José 1 | Charcoal | Beta-554248 | 2.240±30BP | -25.5‰ | acid/alkali/acid | Radiometric PLUS | 390 (25%) 345calBC 323 (70.4%) 205calBC |

**Table B in S1:** List of archaeological sites, location and type of sample analyzed for the stable isotope analysis in this study

| **Site** | **Location** | **Samples** |
| --- | --- | --- |
| Gaioso 13 | Middle Xingu river (VGRX) | Humans |
| Palhal 2 |  | Humans & Fauna |
| Palmeiras |  | Humans &Fauna |
| Pedra do Navio |  | Humans &Fauna |
| Pimental 1 |  | Humans |
| Pimental 2 |  | Humans &Fauna |
| Santo Antônio 1 |  | Humans |
| São José 1 |  | Humans &Fauna |
| Vila Rica 2 |  | Humans &Fauna |
| Bela Vista |  | Humans |
| Santa Luzia |  | Fauna |
| Carrazedo | Xingu river’s delta | Humans &Fauna |

**Table C in S1:** List of human bones and teeth samples analyzed for stable isotope analysis in this study.

| **Site** | **Burial** | **Bone** | **Museum ID** | **Tooth** | **Museum ID** | **Age** | **Sex** | **Museum** |
| --- | --- | --- | --- | --- | --- | --- | --- | --- |
| Gaioso 13 | Urn | Skull | GA-13-Sep.1-01 | Molar NI (M) | GA-13-Sep.1-D1 | Adult | No identified | 1 |
| Palhal 2 | Burial 1 | Femur | PAL-2 – Sep.1-01 | - |  | Adult | Male | 1 |
| Palhal 2 | Burial 2 | Femur | PAL-2 – Sep.2-87 | 3^rd^ right lower molar (3RLM) | PAL-2 – Sep.2-D1 | Adult | Male | 1 |
| Palmeiras | Burial 1 | Left tibia | PA-1 – Sep.1-111 | 3^rd^ right lower molar (3RLM) | PA-1 – Sep.1-D5 | Adult | Male | 1 |
| Palmeiras | Burial 2 | Right tibia | PA-1 – Sep.2-23 | 2^nd^ left upper molar (2LUM) | PA-1 – Sep.2-D2 | Adult | Male | 1 |
| Pedra do Navio | Urn 1 | - | - | 1^st^ lower left Premolar (1LLPM) | PN –  Ur.1-D1 | Adult | No identified | 1 |
| Pedra do Navio | Burial 1 (structure 4) | Left femur |  | 2^nd^ lower left Premolar (2LLPM) | PN –  Sep.1-D1 | Adult | No identified | 1 |
| Pedra do Navio | Burial 2 (structure 6) | Femur | PN – Sep.2-1 | 1^st^ lower right Premolar (1LRPM) | PN –  Sep.2-D1 | Adult | Female | 1 |
| Pedra do Navio | Urn 2 | Skull | PN – Ur.2-1 | - | - | Adult | No identified | 1 |
| Pimental 1 | No identified | Femur | PIM-1 – OI-1 | - | - | Adult | No identified | 1 |
| Pimental 2 | Burial 1 | Left femur | PIM-2 – Sep.1-3 | - | - | Adult | No identified | 1 |
| Pimental 2 | Burial 2 | Femur and humerus | PIM-2 – Sep.2-1 and Sep.2-10 | Molar NI (M) | PI-2 – Sep.2-D2 | Adult | Female | 1 |
| Pimental 2 | Burial 3 | Femur | PIM-2 – Sep.3-2 | - | - | Adult | No identified | 1 |
| Pimental 2 | Burial 4 | Skull | PIM-2 – Sep.4-1.2 | 1^st^ left lower molar (1LLM) | PI-2 – Sep.4-D12.1 | Adult | No identified | 1 |
| Pimental 2 | Burial 5 | Femur | PIM-2 – Sep.5-1 | - | - | Adult | Female | 1 |
| Pimental 2 | Urn 1 | Femur | PIM-2 – Ur.1-1 | - | - | Infant | No identified | 1 |
| Pimental 2 | Urn 2 | Femur | PIM-2 – Ur.2-1 | Molar NI (M) | PI-2 – Ur.2-D1 | Adult | No identified | 1 |
| Santo Antônio 1 | Burial 1 (structure 2) | - | - | Molar NI (M) | SAN-1 – Sep.1-D3 | Possible Infant | No identified | 1 |
| São José 1 | Burial 1 | Skull | SJ-1 – Sep.1-7 | - | - | Adult | No identified | 1 |
| São José 1 | Burial 2 | Left femur | SJ-1 – Sep.2 – 15.1 | 1^st^ right upper molar (1RUM) | SJ-1 – Sep.2 – D21 | Adult | Male | 1 |
| São José 1 | Burial 3 | - | - | Molar NI (M) | SJ-1 – Sep.3 – Dsn | Adult | No identified | 1 |
| São José 1 | Burial 4 | Occipital and Left femur | SJ-1 – Sep.4-21.1 and Sep.4–14 | Premolar NI (PM) | SJ-1 – Sep.4 – D7 | Adult | Female | 1 |
| Vila Rica 2 | Structure 28 | Skull | VR-2 – Ur.28 –1 | 2^nd^ lower molar (2LM) | VR-2 – Ur.28 – D1 | Adult | No identified | 1 |
| Vila Rica 2 | Structure 29 | - | - | Lower Premolar (LPM) | VR-2 – Ur.29 –9 | Adult | No identified | 1 |
| Bela Vista | Urn 1 | Long bone fragment (maybe humerus) | BV-1 – coleta 6 | Molar NI (M) | BV-1 – coleta 4 | Infant | No identified | 2 |
| Carrazedo | Urn 1 | Femur and Tibia | Cr - SN | - | - | Infant | No identified | 2 |
| Total samples | 26 individuals | 25 bones |  | 17 Teeth |  |  |  |  |

1: Sotrage facility Povos do Xingu. Universidade Federal do Pará (UFPA), Altamira, PA. 2: Museu Paraense Emílio Goeldi (MPEG), Belém, PA. The research permit was granted by the Brazilian National Historic and Artistic Heritage Institute (Iphan Process n^o^ 01492.000507/2018-79). “All necessary permits were obtained for the described study, which complies with all pertinent regulations”.

**Table D in S1:** List of samples of fauna bones and teeth analysed for stable isotope analysis in this study

| **Archaeological site** | **Type of sample** | **Common name** | **Taxon** | **N** | **Museum** |
| --- | --- | --- | --- | --- | --- |
| Palhal 2 | Bone | Deer | Mazama sp | 5 | 1 |
| Palhal 2 | Tooth | Deer | Mazama sp | 1 | 1 |
| Palhal 2 | Bone | Alligator | Alligatoridae | 6 | 1 |
| Palhal 2 | Bone | Fish | Osteichthyes | 9 | 1 |
| Palhal 2 | Tooth | Lowland paca | *Cuniculus paca* | 2 | 1 |
| Palhal 2 | Bone | Armadillo | Cingulata | 5 | 1 |
| Palhal 2 | Bone | Tortoise | *Chelonoidis* sp | 7 | 1 |
| Palhal 2 | Bone | Sloth | *Bradypus* sp | 2 | 1 |
| Palhal 2 | Bone | Chelonium | Testudines | 2 | 1 |
| Palhal 2 | Bone | Nine banded armadillo | *Dasypus novemcinctus* | 3 | 1 |
| Palhal 2 | Bone | White-lipped peccary | *Tayassu pecari* | 1 | 1 |
| Palhal 2 | Bone | Reptile | Reptilia | 1 | 1 |
| Palhal 2 | Bone | Bird | Bird | 2 | 1 |
| Palhal 2 | Tooth | Vampire tetra | *Hydrolycus scomberoides* | 1 | 1 |
| Palmeiras 1 | Tooth | Lowland paca | *Cuniculus paca* | 2 | 1 |
| Palmeiras 1 | Bone | Artiodactyla | Artiodactyla | 1 | 1 |
| Palmeiras 1 | Bone | Fish | Osteichthyes | 7 | 1 |
| Palmeiras 1 | Bone | Chelonium | Testudines | 14 | 1 |
| Palmeiras 1 | Bone | Tortoise | *Chelonoidis* sp | 4 | 1 |
| Palmeiras 1 | Bone | Alligator | Alligatoridae | 2 | 1 |
| Palmeiras 1 | Bone | White-lipped peccary | *Tayassu pecari* | 2 | 1 |
| Palmeiras 1 | Bone | Collared peccary | *Pecari tajacu* | 1 | 1 |
| Palmeiras 1 | Bone | Deer | Cervidae | 2 | 1 |
| Palmeiras 1 | Bone | Sloth | Pilosa | 2 | 1 |
| Palmeiras 1 | Bone | Lowland paca | *Cuniculus paca* | 1 | 1 |
| Palmeiras 1 | Bone | Rabbit | *Silvilagus brasiliensis* | 1 | 1 |
| Palmeiras 1 | Tooth | Howler monkey | *Alouatta* | 1 | 1 |
| Palmeiras 1 | Tooth | White-lipped peccary | *Tayassu pecari* | 1 | 1 |
| Palmeiras 1 | Bone | Reptile | Reptilia | 1 | 1 |
| Palmeiras 1 | Bone | Nine-banded armadillo | *Dasypus novemcinctus* | 1 | 1 |
| Palmeiras 1 | Bone | Armadillo | *Cingulata* | 1 | 1 |
| Palmeiras 1 | Bone | Peccary | *Tayassuidae* | 1 | 1 |
| Palmeiras 1 | Tooth | Agouti | *Dasyprocta* sp | 1 | 1 |
| Pimental 2 | Bone | Chelonium | Testudines | 7 | 1 |
| Pimental 2 | Bone | Fish | Osteichthyes | 10 | 1 |
| Pimental 2 | Bone | Deer | *Mazama* sp | 6 | 1 |
| Pimental 2 | Bone | Alligator | Alligatoridae | 6 | 1 |
| Pimental 2 | Bone | Capybara | *Hydrochoerus hydrochaeris* | 2 | 1 |
| Pimental 2 | Bone | Tortoise | *Chelonoidis* sp | 2 | 1 |
| Pimental 2 | Bone | Reptile | Reptilia | 1 | 1 |
| Pimental 2 | Bone | Peccary | *Tayassuidae* | 1 | 1 |
| Pimental 2 | Tooth | Capybara | *Hydrochoerus hydrochaeris* | 1 | 1 |
| Pimental 2 | Bone | Nine-banded armadillo | *Dasypus novemcinctus* | 1 | 1 |
| Pedra do Navio | Bone | Vampire tetra | Osteichthyes | 5 | 1 |
| Pedra do Navio | Tooth | Lowland paca | *Cuniculus paca* | 2 | 1 |
| Pedra do Navio | Tooth | Fish | *Hydrolycus scomberoides* | 1 | 1 |
| Pedra do Navio | Bone | Tortoise | *Chelonoidis* sp | 2 | 1 |
| Pedra do Navio | Tooth | Tapir | *Tapirus* sp | 1 | 1 |
| Pedra do Navio | Bone | Tapir | *Tapirus* sp | 1 | 1 |
| Pedra do Navio | Tooth | Fish (Pacu) | Piaractus mesopotamicus | 2 | 11 |
| Pedra do Navio | Bone | Deer | Cervidae | 1 | 1 |
| Pedra do Navio | Bone | Collared peccary | *Pecari tajacu* | 1 | 1 |
| Santa Luzia 1 | Tooth | Deer | Cervidae | 2 | 1 |
| Santa Luzia 1 | Bone | Peccary | *Tayassuidae* | 2 | 1 |
| Santa Luzia 1 | Bone | Lowland paca | *Cuniculus paca* | 1 | 1 |
| Santa Luzia 1 | Bone | Tortoise | *Chelonoidis* sp | 7 | 1 |
| Santa Luzia 1 | Bone | Tapir | *Tapirus* sp | 6 | 1 |
| Santa Luzia 1 | Tooth | Tapir | *Tapirus* sp | 3 | 1 |
| Santa Luzia 1 | Bone | Deer | Cervidae | 4 | 1 |
| Santa Luzia 1 | Bone | Turtle | Chelonium | 4 | 1 |
| Santa Luzia 1 | Bone | White-lipped peccary | *Tayassu pecari* | 1 | 1 |
| Santa Luzia 1 | Bone | Armadillo | Cingulata | 3 | 1 |
| Santa Luzia 1 | Bone | Giant Armadillo | *Priodontes maximus* | 1 | 1 |
| Santa Luzia 1 | Bone | Fish | Osteichthyes | 5 | 1 |
| Santa Luzia 1 | Tooth | Lowland paca | *Cuniculus paca* | 1 | 1 |
| Santa Luzia 1 | Bone | Tracajá | *Podocnemis unifilis* | 1 | 1 |
| São José 1 | Tooth | White-lipped peccary | *Tayassu pecari* | 1 | 1 |
| São José 1 | Bone | Fish | Osteichthyes | 1 | 1 |
| São José 1 | Tooth | Lowland paca | *Cuniculus paca* | 1 | 1 |
| São José 1 | Bone | Tortoise | *Chelonoidis* sp | 1 | 1 |
| Vila Rica 2 | Bone | Armadillo | Cingulata | 3 | 1 |
| Vila Rica 2 | Tooth | Lowland paca | *Cuniculus paca* | 2 | 1 |
| Vila Rica 2 | Bone | Fish | Osteichthyes | 1 | 1 |
| Carrazedo | Tooth | Lowland paca | *Cuniculus paca* | 1 | 2 |
| Carrazedo | Bone | Lowland paca | *Cuniculus paca* | 1 | 2 |
| Carrazedo | Bone | Turtle | Testudines | 1 | 2 |
| Carrazedo | Bone | Agouti | *Dasyprocta* sp | 2 | 2 |
| Carrazedo | Bone | Armadillo | Cingulata | 1 | 2 |
| Carrazedo | Tooth | Agouti | *Dasyprocta* sp | 1 | 2 |

1: Sotrage facility Povos do Xingu. Universidade Federal do Pará (UFPA), Altamira, PA. 2: Museu Paraense Emílio Goeldi (MPEG), Belém, PA. The research permit was granted by the Brazilian National Historic and Artistic Heritage Institute (Iphan Process n^o^ 01492.000507/2018-79. “All necessary permits were obtained for the described study, complies with all with all pertinent regulations”.

**Table E in S1:** Bulk δ^13^C_co_ and δ^15^N measurements for collagen from pre-colonial fauna from VGRX and mouth, in the Xingu River Basin, Brazil

| **Site** | **Region** | **Species** | **Common name** | **Diet** | **Lab ID** | **Museum ID** | **δ^15^N (‰) (AIR)** | **stdev** | **δ^13^C_co_ (‰) (VPDB)** | **stdev** | **%N** | **%C** | **C/N ratio** | **bone weight** | **collagen weight** | **collagen yield** | **Research** |
| --- | --- | --- | --- | --- | --- | --- | --- | --- | --- | --- | --- | --- | --- | --- | --- | --- | --- |
| Carrazedo | Mouth of Xingu | *Dasyprocta* | Agouti | Herbivorous | CAR002 | SD | 11,24 | 0,02 | -20,49 | 0,11 | 14,17 | 40,28 | 3,3 | 0,674 | 31 | 4,6 | This Research |
| Carrazedo | Mouth of Xingu | *Dasyprocta* | Agouti | Herbivorous | CAR005 | SD | 8,38 | 0,06 | -21,17 | 0,06 | 15,49 | 42,61 | 3,2 | 0,854 | 66 | 7,7 | This Research |
| Carrazedo | Mouth of Xingu | Cuniculus paca | Paca | Herbivorous | CAR007 | SD | 4,97 | 0,04 | -21,99 | 0,14 | 13,97 | 38,11 | 3,2 | 1,437 | 102 | 7,1 | This Research |
| Palhal 2 | VGRX | Osteichthyes | Fish | Fish | PAL019 | PAL 2 - 1275 | 10,43 | 0,03 | -19,70 | 0,09 | 14,47 | 42,69 | 3,4 | 0,485 | 28 | 5,8 | This Research |
| Palhal 2 | VGRX | *Dasypus novemcinctus* | Nine-banded armadillo | Omnivorous | PAL022 | PAL 2 - 1295 | 12,15 | 0,01 | -19,73 | 0,01 | 12,85 | 36,04 | 3,3 | 1,413 | 64 | 4,5 | This Research |
| Palhal 2 | VGRX | Chelonoidis | Tortoise | Omnivorous | PAL023 | PAL 2 - 1296 | 11,55 | 0,03 | -21,73 | 0,06 | 14,01 | 40,78 | 3,4 | 1,333 | 77 | 5,8 | This Research |
| Palhal 2 | VGRX | Chelonoidis | Tortoise | Omnivorous | PAL025 | PAL 2 - 1300 | 11,30 | 0,06 | -21,83 | 0,22 | 14,43 | 40,51 | 3,3 | 1,071 | 52 | 4,9 | This Research |
| Palhal 2 | VGRX | Chelonoidis | Tortoise | Omnivorous | PAL026 | PAL 2 - 1301 | 11,34 | 0,00 | -21,92 | 0,16 | 10,49 | 30,19 | 3,4 | 0,617 | 26 | 4,2 | This Research |
| Palhal 2 | VGRX | Osteichthyes | Fish | Fish | PAL029 | PAL 2 - 1305 | 10,14 | 0,01 | -20,38 | 0,01 | 15,70 | 44,22 | 3,3 | 0,328 | 26 | 7,9 | This Research |
| Palhal 2 | VGRX | *Dasypus novemcinctus* | Nine-banded armadillo | Omnivorous | PAL040 | PAL 2 - 1571 | 11,27 | 0,04 | -20,15 | 0,69 | 14,65 | 42,77 | 3,4 | 0,071 | 5 | 7,0 | This Research |
| Palhal 2 | VGRX | Bird | Bird | Bird | PAL043 | PAL 2 - 1611 | 12,10 | 0,07 | -19,86 | 0,10 | 14,93 | 43,93 | 3,4 | 0,27 | 18 | 6,7 | This Research |
| Palhal 2 | VGRX | Osteichthyes | Fish | Fish | PAL045 | PAL 2 - 1743 | 10,72 | 0,03 | -22,13 | 0,06 | 15,25 | 43,48 | 3,3 | 0,211 | 13 | 6,2 | This Research |
| Palhal 2 | VGRX | Osteichthyes | Fish | Fish | PAL050 | PAL 2 - 1935 | 8,65 | 0,02 | -23,76 | 0,07 | 14,94 | 41,93 | 3,3 | 0,125 | 7 | 5,6 | This Research |
| Palhal 2 | VGRX | Osteichthyes | Fish | Fish | PAL051 | PAL 2 - 1975 | 10,47 | 0,00 | -18,45 | 0,11 | 9,68 | 26,61 | 3,2 | 0,627 | 33 | 5,3 | This Research |
| Palhal 2 | VGRX | Dasypus novemcinctus | Nine-banded armadillo | Omnivorous | PAL054 | PAL 2 - 2051 | 12,09 | 0,01 | -19,53 | 0,04 | 15,19 | 42,51 | 3,3 | 0,396 | 18 | 4,5 | This Research |
| Palmeiras | VGRX | Chelonoidis | Tortoise | Omnivorous | PAM007 | PA 1 - 3 | 11,47 | 0,01 | -22,07 | 0,15 | 10,91 | 31,96 | 3,4 | 1,145 | 84 | 7,3 | This Research |
| Palmeiras | VGRX | Testudinata | Turtle | Omnivorous | PAM015 | PA 1 - 94 | 7,68 | 0,02 | -22,70 | 0,06 | 13,77 | 41,30 | 3,5 | 0,299 | 5 | 1,7 | This Research |
| Palmeiras | VGRX | Pecari tajacu | Collared peccary | Herbivorous | PAM021 | PA 1 - 128 | 10,08 | 0,03 | -21,29 | 0,10 | 8,08 | 23,17 | 3,3 | 1,121 | 59 | 5,3 | This Research |
| Palmeiras | VGRX | Mazama sp. | Deer | Herbivorous | PAM022 | PA 1 - 131 | 8,00 | 0,03 | -21,95 | 0,00 | 13,45 | 38,90 | 3,4 | 1,028 | 30 | 2,9 | This Research |
| Palmeiras | VGRX | Cuniculus paca | Paca | Herbivorous | PAM025 | PA 1 - 212 | 6,60 | 0,01 | -20,38 | 0,10 | 12,92 | 36,32 | 3,3 | 0,873 | 25 | 2,9 | This Research |
| Palmeiras | VGRX | Mazama sp. | Deer | Herbivorous | PAM032 | PA 1 - 372 | 8,57 | 0,03 | -21,65 | 0,02 | 7,07 | 20,83 | 3,4 | 1,207 | 28 | 2,3 | This Research |
| Palmeiras | VGRX | Chelonoidis | Tortoise | Omnivorous | PAM034 | PA 1 - 380 | 11,05 | 0,00 | -22,50 | 0,05 | 9,17 | 26,36 | 3,4 | 1,352 | 57 | 4,2 | This Research |
| Palmeiras | VGRX | *Tayassu pecari* | White-lipped peccary | Herbivorous | PAM039 | PA 1 - 134 | 9,70 | 0,01 | -20,96 | 0,03 | 12,33 | 36,08 | 3,4 | 0,854 | 17 | 2,0 | This Research |
| Palmeiras | VGRX | *Dasypus novemcinctus* | Nine-banded armadillo | Omnivorous | PAM048 | PA 1 - 205 | 13,22 | 0,00 | -19,33 | 0,00 | 14,03 | 40,25 | 3,3 | 0,121 | 3 | 2,5 | This Research |
| Pimental 2 | VGRX | Testudinata | Turtle | Omnivorous | PIM 011 | PI 2 - 131 | 7,73 | 0,01 | -21,86 | 0,09 | 15,76 | 44,55 | 3,3 | 1,583 | 57 | 3,6 | This Research |
| Pimental 2 | VGRX | Testudinata | Turtle | Omnivorous | PIM 016 | PI 2 - 141 | 9,27 | 0,06 | -22,04 | 0,04 | 10,86 | 31,07 | 3,3 | 1,275 | 26 | 2,0 | This Research |
| Pimental 2 | VGRX | Alligatoridae | Alligator | Carnivorous | PIM 018 | PI 2 - 161 | 9,92 | 0,05 | -20,41 | 0,12 | 11,81 | 33,29 | 3,3 | 1,670 | 78 | 4,7 | This Research |
| Pimental 2 | VGRX | Testudinata | Turtle | Omnivorous | PIM 020 | PI 2 - 164 | 8,82 | 0,01 | -22,04 | 0,28 | 9,55 | 26,94 | 3,3 | 1,232 | 71 | 5,8 | This Research |
| Pimental 2 | VGRX | Mazama sp. | Deer | Herbivorous | PIM 027 | PI 2 - 219 | 8,14 | 0,01 | -21,99 | 0,06 | 13,07 | 36,24 | 3,2 | 1,315 | 110 | 8,4 | This Research |
| Pimental 2 | VGRX | Mazama sp. | Deer | Herbivorous | PIM 029 | PI 2 - 237 | 8,19 | 0,02 | -20,92 | 0,21 | 8,10 | 22,72 | 3,3 | 1,425 | 75 | 5,3 | This Research |
| Pimental 2 | VGRX | *Hydrochoerus hydrochaeris* | Capybara | Herbivorous | PIM 030 | PI 2 - 241 | 5,12 | 0,03 | -18,92 | 0,12 | 14,02 | 39,55 | 3,3 | 1,390 | 86 | 6,2 | This Research |
| Pimental 2 | VGRX | Tayassuidae | Peccary | Herbivorous | PIM 031 | PI 2 - 244 | 8,65 | 0,01 | -20,72 | 0,17 | 15,73 | 43,78 | 3,2 | 1,398 | 106 | 7,6 | This Research |
| Pimental 2 | VGRX | *Dasypus novemcinctus* | Nine-banded armadillo | Omnivorous | PIM 035 | PI 2 - 5 | 10,09 | 0,05 | -19,51 | 0,13 | 15,42 | 43,15 | 3,3 | 0,082 | 8 | 9,8 | This Research |
| Pimental 2 | VGRX | Testudinata | Turtle | Omnivorous | PIM001 | PI 2 - 1 | 8,30 | 0,04 | -22,20 | 0,03 | 12,79 | 36,72 | 3,3 | 1,15 | 25 | 2,2 | This Research |
| Pimental 2 | VGRX | Mazama sp. | Deer | Herbivorous | PIM003 | PI 2 - 105 | 9,01 | 0,01 | -22,03 | 0,03 | 11,37 | 33,48 | 3,4 | 1 | 28 | 2,1 | This Research |
| Pimental 2 | VGRX | Testudinata | Turtle | Omnivorous | PIM012 | PI 2 - 132 | 9,20 | 0,03 | -22,65 | 0,21 | 13,06 | 40,62 | 3,6 | 1,341 | 15 | 1,1 | This Research |
| Pimental 2 | VGRX | Testudinata | Turtle | Omnivorous | PIM014 | PI 2 - 137 | 8,85 | 0,06 | -22,51 | 0,09 | 15,28 | 44,46 | 3,4 | 0,78 | 68 | 8,7 | This Research |
| Pimental 2 | VGRX | *Hydrochoerus hydrochaeris* | Capybara | Herbivorous | PIM021 | PI 2 - 172 | 4,90 | 0,02 | -17,94 | 0,02 | 13,29 | 38,72 | 3,4 | 1,348 | 40 | 3,0 | This Research |
| Pimental 2 | VGRX | Alligatoridae | Alligator | Carnivorous | PIM022 | PI 2 - 197 | 10,32 | 0,02 | -20,28 | 0,15 | 11,99 | 36,31 | 3,5 | 0,645 | 15 | 2,3 | This Research |
| Pimental 2 | VGRX | Chelonoidis | Tortoise | Omnivorous | PIM023 | PI 2 - 203 | 8,12 | 0,01 | -22,07 | 0,09 | 14,02 | 40,47 | 3,4 | 1,239 | 65 | 5,2 | This Research |
| Pimental 2 | VGRX | Mazama sp. | Deer | Herbivorous | PIM025 | PI 2 - 211 | 8,20 | 0,03 | -21,93 | 0,23 | 14,34 | 41,59 | 3,4 | 1,318 | 36 | 2,7 | This Research |
| Pimental 2 | VGRX | Osteichthyes | Fish | Fish | PIM032 | PI 2 - 254 | 9,72 | 0,00 | -22,55 | 0,00 | 15,49 | 42,76 | 3,2 | 0,472 | 20 | 4,2 | This Research |
| Pimental 2 | VGRX | Osteichthyes | Fish | Fish | PIM037 | PI 2 - 68 | 8,25 | - | -21,39 | - | 12,75 | 38,65 | 3,5 | 0,026 | 2 | 7,7 | This Research |
| Pedra do Navio | VGRX | *Tapirus terrestris* | Tapir | Herbivorous | PNA014 | PN - 35 | 6,71 | 0,01 | -22,89 | 0,05 | 12,10 | 35,38 | 3,4 | 0,938 | 24 | 2,6 | This Research |
| Santa Luzia 1 | VGRX | *Tapirus terrestris* | Tapir | Herbivorous | SAL 001 | SL 1 - 20 | 9,74 | 0,00 | -23,50 | 0,04 | 9,60 | 27,43 | 3,3 | 1,460 | 72 | 4,9 | This Research |
| Santa Luzia 1 | VGRX | Mazama sp. | Deer | Herbivorous | SAL 002 | SL 1 - 22 | 9,06 | 0,01 | -21,53 | 0,07 | 13,87 | 40,41 | 3,4 | 1,262 | 50 | 4,0 | This Research |
| Santa Luzia 1 | VGRX | Tayassuidae | Peccary | Herbivorous | SAL 008 | SL 1 - 48 | 7,98 | 0,00 | -21,72 | 0,15 | 12,43 | 36,29 | 3,4 | 1,500 | 47 | 3,1 | This Research |
| Santa Luzia 1 | VGRX | *Tapirus terrestris* | Tapir | Herbivorous | SAL 019 | SL 1 - 21 | 9,64 | 0,01 | -23,49 | 0,03 | 13,21 | 37,64 | 3,3 | 1,046 | 31 | 3,0 | This Research |
| Santa Luzia 1 | VGRX | Mazama sp. | Deer | Herbivorous | SAL 020 | SL 1 - 34 | 9,05 | 0,03 | -22,14 | 0,08 | 11,47 | 33,22 | 3,4 | 1,121 | 72 | 6,4 | This Research |
| Santa Luzia 1 | VGRX | *Tapirus terrestris* | Tapir | Herbivorous | SAL004 | SL 1 - 35 | 9,48 | 0,03 | -23,45 | 0,11 | 13,40 | 38,07 | 3,3 | 1,488 | 61 | 4,1 | This Research |
| Santa Luzia 1 | VGRX | Mazama sp. | Deer | Herbivorous | SAL007 | SL 1 - 38 | 8,88 | 0,01 | -21,35 | 0,11 | 16,15 | 44,74 | 3,2 | 1,402 | 112 | 8,0 | This Research |
| Santa Luzia 1 | VGRX | Chelonoidis | Tortoise | Omnivorous | SAL011b | SL 1 - 188 | 12,86 | 0,03 | -23,24 | 0,00 | 12,91 | 37,22 | 3,4 | 2,159 | 21 | 1,0 | This Research |
| Santa Luzia 1 | VGRX | Chelonoidis | Tortoise | Omnivorous | SAL012 | SL 1 - 192 | 12,78 | 0,07 | -23,79 | 0,03 | 3,43 | 10,73 | 3,6 | 1,837 | 18 | 1,0 | This Research |
| Santa Luzia 1 | VGRX | Chelonoidis | Tortoise | Omnivorous | SAL014 | SL 1 - 198 | 13,22 | 0,00 | -22,33 | 0,17 | 14,03 | 40,89 | 3,4 | 1,893 | 47 | 2,5 | This Research |
| Santa Luzia 1 | VGRX | *Tapirus terrestris* | Tapir | Herbivorous | SAL016 | SL 1 - 220 | 7,03 | 0,00 | -23,35 | 0,04 | 10,60 | 30,57 | 3,4 | 1,625 | 16 | 1,0 | This Research |
| Santa Luzia 1 | VGRX | *Tapirus terrestris* | Tapir | Herbivorous | SAL018 | SL 1 - 206 | 7,25 | 0,02 | -23,30 | 0,05 | 14,28 | 39,95 | 3,3 | 1,186 | 52 | 4,4 | This Research |
| Santa Luzia 1 | VGRX | *Tayassu pecari* | White-lipped peccary | Herbivorous | SAL024 | SL 1 - 37 | 8,83 | 0,01 | -22,00 | 0,03 | 14,62 | 41,11 | 3,3 | 1,383 | 111 | 8,0 | This Research |
| Santa Luzia 1 | VGRX | *Priodontes maximus* | Giant Armadillo | Omnivorous | SAL029 | SL 1 - 208 | 13,79 | 0,04 | -21,27 | 0,02 | 3,45 | 10,07 | 3,4 | 1,520 | 15 | 1,0 | This Research |
| Santa Luzia 1 | VGRX | *Tapirus terrestris* | Tapir | Herbivorous | SAL034 | SL 1 - 33 | 9,00 | 0,01 | -23,08 | 0,20 | 15,26 | 43,03 | 3,3 | 1,314 | 91 | 6,9 | This Research |
| Santa Luzia 1 | VGRX | Chelonoidis | Tortoise | Omnivorous | SAL035 | SL 1 - 186 | 12,42 | 0,02 | -23,23 | 0,01 | 5,88 | 18,10 | 3,6 | 1,733 | 30 | 1,7 | This Research |
| Santa Luzia 1 | VGRX | Podocnemis | Tracajá | Omnivorous | SAL046 | SL 1 - 187 | 6,90 | 0,01 | -22,74 | 0,08 | 11,48 | 32,55 | 3,3 | 0,998 | 25 | 2,5 | This Research |

**Table F in S1:** Bulk δ^13^C and δ^18^O measurements for tooth enamel from precolonial human from VGRX, Brazil, as ‰.

| **Site** | **Burial** | **Common name** | **Sex** | **LAB ID** | **Museum ID** | **Tooth** | **δ13Cap (‰) (VPDB)** | **Std. dev.** | **δ18O (‰) (VPDB)** | **Std. dev.** |
| --- | --- | --- | --- | --- | --- | --- | --- | --- | --- | --- |
| Bela Vista | Urn 1 | Human | Not identified | BEL001A | BV-1 – coleta 4 | M | -8,37 | 0,26 | -1,98 | 0,18 |
| Gaioso 13 | Structure 1 | Human | Not identified | GAI001B | GA-13-Sep.1-D1 | M | -11,01 | 0,14 | -3,69 | 0,10 |
| Palhal 2 | Burial 2 | Human | Male | PAL002A | PAL-2 – Sep.2-D1 | 3RLM | -11,94 | 0,13 | -2,93 | 0,10 |
| Palmeiras 1 | Burial 1 | Human | Male | PAM001A | PA-1 – Sep.1-D5 | 3RLM | -11,18 | 0,15 | -3,04 | 0,08 |
| Palmeiras 1 | Burial 2 | Human | Male | PAM002C | PA-1 – Sep.2-D2 | 2LUM | -8,56 | 0,17 | -3,12 | 0,10 |
| Pedra do Navio | Burial 2 (structure 6) | Human | Female | PED003B | PN –  Sep.2-D1 | 1LRPM | -10,62 | 0,19 | -2,83 | 0,11 |
| Pedra do Navio | Urn 1 | Human | Not identified | PED001 | PN –  Ur.1-D1 | 1LLPM | -10,33 | 0,12 | -4,42 | 0,09 |
| Pedra do Navio | Burial 1 (structure 4) | Human | Not identified | PED002B | PN –  Sep.1-D1 | 2LLPM | -8,75 | 0,21 | -2,87 | 0,06 |
| Pimental 2 | Urn 2 | Human | Not identified | PIM007B | PI-2 – Ur.2-D1 | M | -10,42 | 0,18 | -4,01 | 0,14 |
| Pimental 2 | Burial 2 | Human | Female | PIM002C | PI-2 – Sep.2-D2 | M | -10,02 | 0,18 | -2,79 | 0,08 |
| Pimental 2 | Burial 4 | Human | Not identified | PIM004B | PI-2 – Sep.4-D12.1 | 1LLM | -10,01 | 0,16 | -2,96 | 0,13 |
| Santo Antônio 1 | Urn 2 | Human | Not identified | SAN001B | SAN-1 – Sep.1-D3 | 1LLM | -12,06 | 0,17 | -3,36 | 0,16 |
| São José 1 | Burial 3 | Human | Not identified | SAO003 | SJ-1 –  Sep.3-Dsn | M | -13,27 | 0,14 | -3,07 | 0,08 |
| São José 1 | Burial 2 | Human | Male | SAO002A | SJ-1 – Sep.2 – D21 | 1RUM | -11,35 | 0,14 | -4,14 | 0,12 |
| São José 1 | Burial 4 | Human | Female | SAO004A | SJ-1 – Sep.4 – D7 | PM | -10,56 | 0,21 | -3,17 | 0,09 |
| Vila Rica 2 | Structure 28 | Human | Not identified | VIL001A | VR-2 – Ur.28 – D1 | 2LM | -10,17 | 0,15 | -2,92 | 0,15 |
| Vila Rica 2 | Structure 29 | Human | Not identified | VIL002B | VR-2 – Ur.29 –9 | LPM | -8,73 | 0,18 | -2,35 | 0,13 |

**Table G in S1:** Bulk δ^13^C and δ^18^O measurements for tooth enamel from precolonial fauna from VGRX and mouth of the Xingu River Basin, Brazil, as ‰.

| **Site** | **Common name** | **Taxon** | **Diet** | **δ13Cap (‰) (VPDB)** | **LAB ID** | **Std. dev.** | **δ18O (‰) (VPDB)** | **Std. dev.** |
| --- | --- | --- | --- | --- | --- | --- | --- | --- |
| Pedra do Navio | Fish (Vampire tetra) | *Hydrolycus scomberoides* | Fish (Carnivorous) | -13.1 |  | 0.2 | -3.6 | 0.2 |
| Palhal 2 | Fish (Vampire tetra) | *Hydrolycus scomberoides* | Fish (Carnivorous) | -11.2 |  | 0.3 | -6.2 | 0.4 |
| Carrazedo (Mouth Xingu) | Agouti | *Dasyprocta* sp | Herbivorous | -15.4 |  | 0.2 | -3.8 | 0.1 |
| Palmeiras | Agouti | *Dasyprocta* sp | Herbivorous | -13.7 |  | 0.2 | -5.6 | 0.1 |
| Pimental 2 | Capybara | *Hydrochoerus hydrochaeris* | Herbivorous | -13.7 |  | 0.1 | -4.7 | 0.1 |
| Santa Luzia 1 | Deer | Mazama sp. | Herbivorous | -14.8 |  | 0.1 | -2.3 | 0.1 |
| Santa Luzia 1 | Deer | Mazama sp. | Herbivorous | -15.2 |  | 0.1 | -3.6 | 0.1 |
| Palhal 2 | Deer | Mazama sp. | Herbivorous | -13.2 |  | 0.2 | -3.2 | 0.2 |
| Palmeiras | Howler monkey | *Alouatta* | Herbivorous | -15.4 |  | 0.1 | -2.7 | 0.2 |
| Carrazedo (Mouth Xingu) | Lowland paca | *Cuniculus paca* | Herbivorous | -15.5 |  | 0.2 | -3.5 | 0.1 |
| Palhal 2 | Lowland paca | *Cuniculus paca* | Herbivorous | -15.3 |  | 0.2 | -6.5 | 0.1 |
| Palhal 2 | Lowland paca | *Cuniculus paca* | Herbivorous | -15.1 |  | 0.3 | -5.8 | 0.1 |
| Palmeiras | Lowland paca | *Cuniculus paca* | Herbivorous | -13.3 |  | 0.2 | -6.1 | 0.2 |
| Palmeiras | Lowland paca | *Cuniculus paca* | Herbivorous | -14.1 |  | 0.2 | -2.9 | 0.2 |
| Pedra do Navio | Lowland paca | *Cuniculus paca* | Herbivorous | -15.5 |  | 0.2 | -4.6 | 0.2 |
| Pedra do Navio | Lowland paca | *Cuniculus paca* | Herbivorous | -14.8 |  | 0.2 | -3.4 | 0.1 |
| Santa Luzia 1 | Lowland paca | *Cuniculus paca* | Herbivorous | -15.4 |  | 0.2 | -5.6 | 0.2 |
| São José 1 | Lowland paca | *Cuniculus paca* | Herbivorous | -14.9 |  | 0.2 | -4.8 | 0.1 |
| Vila Rica 2 | Lowland paca | *Cuniculus paca* | Herbivorous | -14.5 |  | 0.2 | -2.2 | 0.1 |
| Vila Rica 2 | Lowland paca | *Cuniculus paca* | Herbivorous | -15.7 |  | 0.2 | -3.6 | 0.1 |
| Santa Luzia 1 | Tapir | *Tapirus* sp | Herbivorous | -15.4 |  | 0.1 | -3.4 | 0.1 |
| Pedra do Navio | Tapir | *Tapirus* sp | Herbivorous | -15.1 |  | 0.2 | -6.7 | 0.1 |
| Santa Luzia 1 | Tapir | *Tapirus* sp | Herbivorous | -14.8 |  | 0.2 | -4.9 | 0.1 |
| Santa Luzia 1 | Tapir | *Tapirus* sp | Herbivorous | -16.4 |  | 0.2 | -2.3 | 0.2 |
| Pedra do Navio | Fish (Pacu) | Myleinae | Fish (Omnivorous) | -10.7 |  | 0.2 | -6.1 | 0.2 |
| Pedra do Navio | Fish (Pacu) | Myleinae | Fish (Omnivorous) | -9.5 |  | 0.3 | -4.3 | 0.2 |
| Palmeiras | White lipped peccary | *Tayassu pecari* | Herbivorous | -15.0 |  | 0.1 | -4.0 | 0.1 |
| São José 1 | White lipped peccary | *Tayassu pecari* | Herbivorous | -13.0 |  | 0.1 | -4.1 | 0.1 |

**Table H in S1:** Bulk δ^13^C_co_ and δ^15^N measurements of pre-colonial humans from archaeological sites from this study and published studies in the Amazon Basin

| **Site/Tradition** | **δ^13^C ‰VPDB** | **δ^15^N ‰AIR** | **Ratio C/N** | **Context** |
| --- | --- | --- | --- | --- |
| Palmeiras 1 | -16.3 | 11.3 | 3.3 | VGRX^1^ |
| Palmeiras 1 | -16.7 | 12 | 3.5 | VGRX^1^ |
| Palhal 2 | -18.2 | 13.2 | 3.2 | VGRX^1^ |
| Palhal 2 | -16 | 12.4 | 2.9 | VGRX^1^ |
| Gruta das Caretas | -18.9 | 11.5 | 3.3 | Maracá^2^ |
| Gruta das Caretas | -19 | 10.9 | 3.4 | Maracá^2^ |
| Gruta das Caretas | -18.3 | 11.3 | 3.5 | Maracá^2^ |
| Gruta das Caretas | -17.8 | 11.9 | 3.3 | Maracá^2^ |
| Gruta das Caretas | -18.2 | 11.3 | 3.3 | Maracá^2^ |
| Gruta das Caretas | -18.8 | 11.9 | 3.6 | Maracá^2^ |
| Gruta das Caretas | -20.6 | 10.6 | 3.6 | Maracá^2^ |
| Gruta das Caretas | -18.2 | 11.6 | 3.3 | Maracá^2^ |
| Gruta das Caretas | -18.2 | 11.9 | 3.3 | Maracá^2^ |
| Gruta das Caretas | -18.2 | 11.7 | 3.3 | Maracá^2^ |
| Gruta das Caretas | -18.5 | 12 | 3.5 | Maracá^2^ |
| Gruta das Caretas | -22.1 | 10.4 | 3.4 | Maracá^2^ |
| Gruta das Caretas | -18.9 | 11.3 | 3.2 | Maracá^2^ |
| Gruta das Caretas | -19.6 | 11 | 3.6 | Maracá^2^ |
| Gruta das Caretas | -19 | 11.3 | 3.6 | Maracá^2^ |
| Gruta do Pocinho | -19.8 | 11.8 | 3.4 | Maracá^2^ |
| Gruta do Pocinho | -18.4 | 11.6 | 3.4 | Maracá^2^ |
| Unknown | -18.1 | 12.2 | n/a | Marajoara^2^ |
| Cachoeira | -16.3 | 10.5 | n/a | Marajoara^2^ |
| Teso dos Bichos | -14.3 | 7.6 | n/a | Marajoara^2^ |
| Unknown | -16.9 | 9.9 | n/a | Marajoara^2^ |
| Unknown | -17.8 | 10.4 | n/a | Marajoara^2^ |
| Monte Carmelo | -17.9 | 10.7 | n/a | Marajoara^2^ |
| Matinadas | -15.8 | 9.4 | n/a | Marajoara^2^ |
| Late Corazal | -9.7 | n/a | n/a | Corazal^3^ |
| Late Corazal | -10.5 | n/a | n/a | Corazal^3^ |
| Late Corazal | -10.8 | n/a | n/a | Corazal^3^ |
| Early Corazal | -26.1 | n/a | n/a | Corazal^3^ |
| Early Corazal | -25.8 | n/a | n/a | Corazal^3^ |
| Shahuaya | -10.3 | 7.5 | n/a | Ucayali^4^ |
| Shahuaya | -11.7 | 8 | n/a | Ucayali^4^ |
| Shahuaya | -11.8 | 8.7 | n/a | Ucayali^4^ |
| Shahuaya | -12.1 | 7.9 | n/a | Ucayali^4^ |
| Shahuaya | -11.7 | 10.8 | n/a | Ucayali^4^ |
| Nueva Esperanza | -14.6 | 10.1 | n/a | Ucayali^4^ |
| Olaya | -13.6 | 9 | n/a | Ucayali^4^ |
| Olaya | -15.3 | 10.9 | n/a | Ucayali^4^ |
| Olaya | -15.4 | 10.6 | n/a | Ucayali^4^ |
| Mina | -18.3 | 12.2 | 3.3 | São Luis^5^ |
| Mina/Incised Rim | -17.5 | 12.3 | 3.2 | São Luis^5^ |
| Polychome | -16.5 | 11.1 | 3.4 | São Luis^5^ |
| Polychome | -16.4 | 12.4 | 3.3 | São Luis^5^ |
| Mina | -16.4 | 12.4 | 3.2 | São Luis^5^ |

1- This research; 2- Hermenegildo et al (37); 3- Van den Merwe et al. (38); 4- Roosevelt (39); 5- Colonese et al. (40).

**Table I in S1:** Pairwise comparisons using Wilcoxon rank sum test for δ^13^C_co_ of fauna from VGRX by dietary groups.

|  | **Carnivorous** | **Fish** | **Herbivorous** |
| --- | --- | --- | --- |
| **Fish** | 1.00 | - | - |
| **Herbivorous** | 0.39 | 1.00 | - |
| **Omnivorous** | 0.97 | 1.00 | 1.00 |

**Table J in S1:** Results of ANOVA for δ^15^N of fauna from VGRX by dietary group and taxa.

|  | **Df** | **Sum Sq** | **Mean Sq** | **F value** | **Pr(>F)** |
| --- | --- | --- | --- | --- | --- |
| **Group** | 3 | 75.58 | 25.192 | 22.226 | 5.60e-09 *** |
| **Taxa** | 11 | 114.74 | 10.430 | 9.202 | 2.47e-08 *** |
| **Residuals** | 45 | 51.01 | 1.133 |  |  |

**Table K in S1:** Results of post-Hoc Tukey pairwise comparison for δ^15^N of fauna from VGRX by dietary group. 95% confidence interval of difference is indicated alongside 95% probability of lower and upper bounds of this difference.

|  | **Difference** | **Lower** | **Upper** | ***p*-value*** |
| --- | --- | --- | --- | --- |
| **Fish-Carnivorous** | -0.3514286 | --2.6286188 | 1.9257617 | 0.9761778 |
| **Herbivorous-Carnivorous** | -1.8829630 | -3.9643086 | 0.1983826 | 0.0889756 |
| **Omnivorous-Carnivorous** | 0.5245833 | -1.5657143 | 2.6148810 | 0.9080327 |
| **Herbivorous-Fish** | -1.5315344 | -2.7361560 | -0.3269128 | 0.0076722* |
| **Omnivorous-Fish** | 0.8760119 | -0.3440119 | 2.0960357 | 0.2361393 |
| **Omnivorous-Herbivorous** | 2.4075463 | 1.6107640 | 3.2043286 | 0.0000000* |

**Table L in S1**: Pairwise comparisons using Wilcoxon rank sum test for δ^13^C_ap_ of fauna from VGRX by diet

|  | **Fish (Carnivorous)** | **Fish (Omnivorous)** |
| --- | --- | --- |
| **Fish (Omnivorous)** | 0.245 | - |
| **Herbivorous** | 0.071 | 0.071 |

**Table M in S1:** Results of ANOVA for δ^18^O of faunal tooth enamel from VGRX by dietary group and taxa.

|  | | | |  |  |
| --- | --- | --- | --- | --- | --- |
|  | **Df** | **Sum Sq** | **Mean Sq** | **F value** | **Pr(>F)** |
| **Diet** | 2 | 2.61 | 1.303 | 0.635 | 0.541 |
| **Common name** | 6 | 8.08 | 1.347 | 0.656 | 0.685 |
| **Residuals** | 19 | 39.01 | 2.053 |  |  |

**Table N in S1:** Results of post-Hoc Tukey pairwise comparison for δ^18^O of fauna from VGRX by dietary group. 95% confidence interval of difference is indicated alongside 95% probability of lower and upper bounds of this difference.

|  | **Difference** | **Lower** | **Upper** | ***p*-value*** |
| --- | --- | --- | --- | --- |
| **Fish (Omnivorous)-Fish (Carnivorous)** | -0.2500000 | -3.890227 | 3.390227 | 0.9833733 |
| **Herbivorous-Fish (Carnivorous)** | 0.73625 | -1.942885 | 3.415385 | 0.7674373 |
| **Herbivorous-Fish (Omnivorous)** | 0.98625 | -1.692885 | 3.665385 | 0.625334 |

**Table O in S1:** Pairwise comparisons using Wilcoxon rank sum test for δ^13^C_ap_ of fauna from VGRX (archaeological), Ucayali (archaeological) and Modern samples from Peru (including Capybara)

|  | **Modern** | **Ucayali (archaeological)** |
| --- | --- | --- |
| **Ucayali (archaeological)** | 0.00037* | - |
| **VGRX** | 0.00709* | 0.00167* |

**Table P in S1**: Pairwise comparisons using Wilcoxon rank sum test for δ^13^C_ap_ of fauna from VGRX (archaeological), Ucayali (archaeological) and Modern samples from Peru (excluding Capybara)

|  | **Modern** | **Ucayali (archaeological)** |
| --- | --- | --- |
| **Ucayali (archaeological)** | 0.0007* | - |
| **VGRX** | 0.0047* | 0.0047* |

**Table Q in S1:** Pairwise comparisons using Wilcoxon rank sum test for δ^13^C_co_ of archaeological fauna from VGRX and Ucayali by Taxa.

|  | **Capybara Ucayali** | **Capybara VGRX** | **Deer Ucayali** | **Deer VGRX** | **Fish Ucayali** | **Fish VGRX** | **Paca Ucayali** | **Paca VGRX** | **Peccary Ucayali** |
| --- | --- | --- | --- | --- | --- | --- | --- | --- | --- |
| **Capybara VGRX** | 1.0 | - | - | - | - | - | - | - | - |
| **Deer Ucayali** | 1.0 | 1.0 | - | - | - | - | - | - | - |
| **Deer VGRX** | 1.0 | 1.0 | 0.7 | - | - | - | - | - | - |
| **Fish Ucayali** | 1.0 | 1.0 | 1.0 | 0.3 | - | - | - | - | - |
| **Fish VGRX** | 1.0 | 1.0 | 1.0 | 1.0 | 1.0 | - | - | - | - |
| **Paca Ucayali** | 1.0 | 1.0 | 1.0 | 1.0 | 1.0 | 1.0 | - | - | - |
| **Paca VGRX** | 1.0 | 1.0 | 1.0 | 1.0 | 1.0 | 1.0 | 1.0 | - | - |
| **Peccary Ucayali** | 1.0 | 1.0 | 1.0 | 1.0 | 1.0 | 1.0 | 1.0 | 1.0 | - |
| **Peccary VGRX** | 1.0 | 1.0 | 1.0 | 1.0 | 1.0 | 1.0 | 1.0 | 1.0 | 1.0 |

**Table R in S1:** Results of ANOVA for δ^15^N of archaeological fauna from VGRX and Ucayali by Taxa.

|  | **Df** | **Sum Sq** | **Mean Sq** | **F value** | **Pr(>F)** |
| --- | --- | --- | --- | --- | --- |
| **Taxa** | 8 | 103.6 | 12.951 | 8.469 | 2.04e-05 |
| R**esiduals** | 24 | 36.7 | 1.529 |  |  |

**Table S in S1:** Results of post-Hoc Tukey pairwise comparison for δ^15^N of archaeological fauna from VGRX and Ucayali by Taxa. 95% confidence interval of difference is indicated alongside 95% probability of lower and upper bounds of this difference.

|  | **Difference** | **Lower** | **Upper** | ***p*-value*** |
| --- | --- | --- | --- | --- |
| **Capybara VGRX-Capybara Ucayali** | 1.400000e+00 | -3.74767464 | 6.5476746 | 0.9891295 |
| **Deer Ucayali-Capybara Ucayali** | 2.100000e+00 | -2.75327419 | 6.9532742 | 0.8579199 |
| **Deer VGRX-Capybara Ucayali** | 4.970000e+00 | 0.53958708 | 9.4004129 | 0.0198588* |
| **Fish Ucayali-Capybara Ucayali** | 4.233333e+00 | -0.61994086 | 9.0866075 | 0.1221671 |
| **Fish VGRX-Capybara Ucayali** | 6.157143e+00 | 1.66388406 | 10.6504017 | 0.0026768* |
| **Paca Ucayali-Capybara Ucayali** | 2.150000e+00 | -2.54916253 | 6.8491625 | 0.8185201 |
| **Peccary Ucayali-Capybara Ucayali** | 2.100000e+00 | -3.04767464 | 7.2476746 | 0.8921224 |
| **Peccary VGRX-Capybara Ucayali** | 4.755000e+00 | -0.39267464 | 9.9026746 | 0.0862144 |
| **Deer Ucayali-Capybara VGRX** | 7.000000e-01 | -3.13685014 | 4.5368501 | 0.9992907 |
| **Deer VGRX-Capybara VGRX** | 3.570000e+00 | 0.28431784 | 6.8556822 | 0.0260902* |
| **Fish Ucayali-Capybara VGRX** | 2.833333e+00 | -1.00351680 | 6.6701835 | 0.2758299 |
| **Fish VGRX-Capybara VGRX** | 4.757143e+00 | 1.38719876 | 8.1270870 | 0.0019029* |
| **Paca Ucayali-Capybara VGRX** | 7.500000e-01 | -2.88995564 | 4.3899556 | 0.9983139 |
| **Peccary Ucayali-Capybara VGRX** | 7.000000e-01 | -3.50305874 | 4.9030587 | 0.9996346 |
| **Peccary VGRX-Capybara VGRX** | 3.355000e+00 | -0.84805874 | 7.5580587 | 0.1952572 |
| **Deer VGRX-Deer Ucayali** | 2.870000e+00 | 0.06796084 | 5.6720392 | 0.0417961* |
| **Fish Ucayali-Deer Ucayali** | 2.133333e+00 | -1.29844976 | 5.5651164 | 0.4880461 |
| **Fish VGRX-Deer Ucayali** | 4.057143e+00 | 1.15675678 | 6.9575289 | 0.0021154* |
| **Paca Ucayali-Deer Ucayali** | 5.000000e-02 | -3.16013914 | 3.2601391 | 1.0000000 |
| **Peccary Ucayali-Deer Ucayali** | 4.440892e-15 | -3.83685014 | 3.8368501 | 1.0000000 |
| **Peccary VGRX-Deer Ucayali** | 2.655000e+00 | -1.18185014 | 6.4918501 | 0.3523713 |
| **Fish Ucayali-Deer VGRX** | -7.366667e-01 | -3.53870583 | 2.0653725 | 0.9912539 |
| **Fish VGRX-Deer VGRX** | 1.187143e+00 | -0.93099965 | 3.3052854 | 0.6169844 |
| **Paca Ucayali-Deer VGRX** | -2.820000e+00 | -5.34572397 | -0.2942760 | 0.0206924* |
| **Peccary Ucayali-Deer VGRX** | -2.870000e+00 | -6.15568216 | 0.4156822 | 0.1211831 |
| **Peccary VGRX-Deer VGRX** | -2.150000e-01 | -3.50068216 | 3.0706822 | 0.9999997 |
| **Fish VGRX-Fish Ucayali** | 1.923810e+00 | -0.97657656 | 4.8241956 | 0.4051901 |
| **Paca Ucayali-Fish Ucayali** | -2.083333e+00 | -5.29347247 | 1.1268058 | 0.4329285 |
| **Peccary Ucayali-Fish Ucayali** | -2.133333e+00 | -5.97018347 | 1.7035168 | 0.6264163 |
| **Peccary VGRX-Fish Ucayali** | 5.216667e-01 | -3.31518347 | 4.3585168 | 0.9999194 |
| **Paca Ucayali-Fish VGRX** | -4.007143e+00 | -6.64154934 | -1.3727364 | 0.0007682* |
| **Peccary Ucayali-Fish VGRX** | -4.057143e+00 | -7.42708695 | -0.6871988 | 0.0103740* |
| **Peccary VGRX-Fish VGRX** | -1.402143e+00 | -4.77208695 | 1.9678012 | 0.8814800 |
| **Peccary Ucayali-Paca Ucayali** | -5.000000e-02 | -3.68995564 | 3.5899556 | 1.0000000 |
| **Peccary VGRX-Paca Ucayali** | 2.605000e+00 | -1.03495564 | 6.2449556 | 0.3118741 |
| **Peccary VGRX-Peccary Ucayali** | 2.655000e+00 | -1.54805874 | 6.8580587 | 0.4675191 |

**Table T in S1:** Pairwise comparisons using Wilcoxon rank sum test for δ^13^C_co_ human remains from this and published studies in the Amazon Basin

|  | **Early Corazal** | **Early Ucayali** | **Late Corazal** | **Late Ucayali** | **Maracá** | **Marajoara** | **São Luís** |
| --- | --- | --- | --- | --- | --- | --- | --- |
| **Early Ucayali** | 0.736 | - | - | - | - | - | - |
| **Late Corazal** | 0.736 | 0.622 | - | - | - | - | - |
| **Late Ucayali** | 0.708 | 0.389 | 0.736 | - | - | - | - |
| **Maracá** | 0.441 | 0.067 | 0.173 | 0.026* | - | - | - |
| **Marajoara** | 0.622 | 0.614 | 0.389 | 0.136 | 0.009* | - | - |
| **São Luís** | 0.708 | 0.389 | 0.501 | 0.245 | 0.102 | 1.000 | - |
| **VGRX** | 0.736 | 0.456 | 0.622 | 0.389 | 0.136 | 1.000 | 0.736 |

**Table U in S1:** Pairwise comparisons using Wilcoxon rank sum test for δ^15^N human remains from this and published studies in the Amazon Basin

|  | **Early Ucayali** | **Late Ucayali** | **Maracá** | **Marajoara** | **São Luis** |
| --- | --- | --- | --- | --- | --- |
| **Late Ucayali** | 0.557 | - | - | - | - |
| **Maracá** | 0.110 | 0.025* | - | - | - |
| **Marajoara** | 1.000 | 0.776 | 0.156 | - | - |
| **São Luis** | 0.195 | 0.150 | 0.195 | 0.150 | - |
| **VGRX** | 0.208 | 0.195 | 0.776 | 0.208 | 1.000 |

**Table V in S1:** Bulk δ^13^C_co_ and δ^15^N measurements for collagen from pre-colonial human from VGRX, in the Xingu River Basin, Brazil

| **Site** | **Region** | **Burial** | **Common name** | **Sex** | **Lab ID** | **δ^15^N (‰) (AIR)** | **stdev** | **δ^13^C_co_ (‰) (VPDB)** | **stdev** | **%N** | **%C** | **C/N ratio** | **bone weight** | **collagen weight** | **collagen yield** | **Research** |
| --- | --- | --- | --- | --- | --- | --- | --- | --- | --- | --- | --- | --- | --- | --- | --- | --- |
| Palhal 2 | VGRX | Burial 1 | Human | Male | PAL001B | 13.23 | 0.05 | -18.18 | 0.01 | 13.37 | 36.51 | 3.2 | 1.298 | 0.017 | 1.3 | This Research |
| Palhal 2 | VGRX | Burial 2 | Human | Male | PAL002B | 12.40 | 0.04 | -16.00 | 0.04 | 18.09 | 44.79 | 2.9 | 1.127 | 0.077 | 6.8 | This Research |
| Palmeiras | VGRX | Burial 1 | Human | Male | PAM001B | 11.28 | 0.08 | -16.31 | 0.01 | -16.31 | 0.01 | 3.3 | 1.261 | 0.042 | 3.3 | This Research |
| Palmeiras | VGRX | Burial 2 | Human | Male | PAM002A | 11.51 | 0.02 | -15.69 | 0.02 | 17.55 | 44.62 | 3.0 | 1.28 | 0.041 | 3.2 | This Research |

**Table W in S1:** Herbivorous fauna extracted from Roosevelt (39) used in this study for δ^13^C_ap_ comparison.

| **Commun nome** | **Taxon** | **δ^13^Cap ‰VPDB** | **Samples** |
| --- | --- | --- | --- |
| Deer | *Odocoileus* sp. | -13,7 | Ucayali (archaeological) |
| Deer | *Odocoileus* sp. | -13,2 | Ucayali (archaeological) |
| Deer | *Odocoileus* sp. | -13,3 | Ucayali (archaeological) |
| Capybara | *Hydrochoerus hydrochaeris* | -8,7 | Ucayali (archaeological) |
| Capybara | *Hydrochoerus hydrochaeris* | -8,5 | Ucayali (archaeological) |
| Paca | *Canuculus paca* | -15,9 | Ucayali (archaeological) |
| Paca | *Canuculus paca* | -13,9 | Ucayali (archaeological) |
| Paca | *Canuculus paca* | -13 | Ucayali (archaeological) |
| Paca | *Canuculus paca* | -10,8 | Ucayali (archaeological) |
| Peccary | *Tayassu* sp. | -14,1 | Ucayali (archaeological) |
| Peccary | *Tayassu* sp. | -12 | Ucayali (archaeological) |
| Peccary | *Tayassu* sp. | -12,9 | Ucayali (archaeological) |

**Table X in S1:** Modern herbivorous fauna extracted from Tejada et.al. (29) used in this study for δ^13^C_ap_ comparison. Tejada et al analysed a greater variety of species and diets, such as insectivores, carnivores and omnivores. For comparisons, we used only herbivorous and fruit-eating species, as we do not have carnivores and omnivores in our samples.

| **Commun nome** | **Taxon** | **δ^13^Cap ‰VPDB** **_1750** | **Samples** |
| --- | --- | --- | --- |
| red brocket | *Mazama americana* | -12.6 | Modern |
| red brocket | *Mazama americana* | -13.1 | Modern |
| red brocket | *Mazama americana* | -15.0 | Modern |
| red brocket | *Mazama americana* | -13.6 | Modern |
| gray/brown brocket | *Mazama gouazoubira* | -13.0 | Modern |
| gray/brown brocket | *Mazama gouazoubira* | -12.4 | Modern |
| gray/brown brocket | *Mazama gouazoubira* | -12.6 | Modern |
| collared pecari | *Pecari tajacu* | -13.2 | Modern |
| collared pecari | *Pecari tajacu* | -12.2 | Modern |
| collared pecari | *Pecari tajacu* | -13.0 | Modern |
| collared pecari | *Pecari tajacu* | -12.5 | Modern |
| white-lipped pecari | *Tayassu pecari* | -12.6 | Modern |
| white-lipped pecari | *Tayassu pecari* | -13.4 | Modern |
| white-lipped pecari | *Tayassu pecari* | -14.1 | Modern |
| white-lipped pecari | *Tayassu pecari* | -13.5 | Modern |
| white-lipped pecari | *Tayassu pecari* | -13.6 | Modern |
| kinkajou | *Potos flavus* | -16.1 | Modern |
| kinkajou | *Potos flavus* | -16.0 | Modern |
| kinkajou | *Potos flavus* | -16.4 | Modern |
| kinkajou | *Potos flavus* | -16.2 | Modern |
| kinkajou | *Potos flavus* | -16.4 | Modern |
| forest rabbit | *Sylvilagus brasiliensis* | -12.7 | Modern |
| forest rabbit | *Sylvilagus brasiliensis* | -17.7 | Modern |
| forest rabbit | *Sylvilagus brasiliensis* | -17.3 | Modern |
| lowland tapir | *Tapirus terrestris* | -15.9 | Modern |
| lowland tapir | *Tapirus terrestris* | -16.5 | Modern |
| lowland tapir | *Tapirus terrestris* | -15.5 | Modern |
| lowland tapir | *Tapirus terrestris* | -17.5 | Modern |
| lowland tapir | *Tapirus terrestris* | -14.6 | Modern |
| lowland tapir | *Tapirus terrestris* | -14.9 | Modern |
| lowland tapir | *Tapirus terrestris* | -15.7 | Modern |
| red howler monkey | *Alouatta seniculus* | -15.6 | Modern |
| red howler monkey | *Alouatta seniculus* | -16.6 | Modern |
| red howler monkey | *Alouatta seniculus* | -16.4 | Modern |
| red howler monkey | *Alouatta seniculus* | -16.8 | Modern |
| Peruvian nigh monkey | *Aotus nigriceps* | -17.2 | Modern |
| Peruvian nigh monkey | *Aotus nigriceps* | -14.7 | Modern |
| Peruvian nigh monkey | *Aotus nigriceps* | -16.0 | Modern |
| night monkey | *Aotus nigriceps* | -16.5 | Modern |
| night monkey | *Aotus nigriceps* | -17.2 | Modern |
| spider monkey | *Ateles chamek* | -16.8 | Modern |
| spider monkey | *Ateles chamek* | -15.2 | Modern |
| spider monkey | *Ateles chamek* | -16.9 | Modern |
| brown titi monkey | *Callicebus brunneus* | -18.3 | Modern |
| brown titi monkey | *Callicebus brunneus* | -17.7 | Modern |
| red titi monkey | *Plecturocebus discolor* | -19.3 | Modern |
| coppery titi monkey | *Callicebus cupreus* | -17.0 | Modern |
| coppery titi monkey | *Callicebus cupreus* | -16.1 | Modern |
| brown titi monkey | *Callicebus urubambensis* | -18.2 | Modern |
| brown titi monkey | *Callicebus urubambensis* | -17.3 | Modern |
| white-fronted capuchin | *Cebus albifrons* | -16.8 | Modern |
| white-fronted capuchin | *Cebus albifrons* | -17.8 | Modern |
| white-fronted capuchin | *Cebus albifrons* | -17.5 | Modern |
| white-fronted capuchin | *Cebus albifrons* | -17.1 | Modern |
| white-fronted capuchin | *Cebus albifrons* | -18.0 | Modern |
| white-fronted capuchin | *Cebus albifrons* | -17.5 | Modern |
| white-fronted capuchin | *Cebus albifrons* | -17.9 | Modern |
| brown capuchin | *Sapaius apella* | -16.8 | Modern |
| brown capuchin | *Sapaius apella* | -17.7 | Modern |
| brown capuchin | *Sapaius apella* | -15.3 | Modern |
| brown capuchin | *Sapaius apella* | -16.5 | Modern |
| brown capuchin | *Sapaius apella* | -17.7 | Modern |
| Peruvian woolly monkey | *Lagothrix lagotricha* | -15.9 | Modern |
| woolly monkey | *Lagothrix lagotricha* | -16.3 | Modern |
| woolly monkey | *Lagothrix lagotricha* | -15.2 | Modern |
| woolly monkey | *Lagothrix lagotricha* | -16.6 | Modern |
| woolly monkey | *Lagothrix lagotricha* | -16.8 | Modern |
| woolly monkey | *Lagothrix lagotricha* | -16.5 | Modern |
| woolly monkey | *Lagothrix lagotricha* | -16.0 | Modern |
| woolly monkey | *Lagothrix lagotricha* | -15.4 | Modern |
| woolly monkey | *Lagothrix lagotricha* | -16.7 | Modern |
| woolly monkey | *Lagothrix lagotricha* | -16.5 | Modern |
| woolly monkey | *Lagothrix lagotricha* | -15.9 | Modern |
| woolly monkey | *Lagothrix lagotricha* | -15.6 | Modern |
| saddleback tamarin | *Saguinus fuscicollis* | -16.5 | Modern |
| saddleback tamarin | *Saguinus fuscicollis* | -16.4 | Modern |
| saddleback tamarin | *Saguinus fuscicollis* | -16.0 | Modern |
| agouti | *Cuniculus paca* | -15.3 | Modern |
| agouti | *Cuniculus paca* | -15.8 | Modern |
| agouti | *Cuniculus paca* | -15.5 | Modern |
| bicolor-spined porcupine | *Coendou bicolor* | -13.1 | Modern |
| bicolor-spined porcupine | *Coendou bicolor* | -11.8 | Modern |
| Brazilian porcupine | *Coendou prehensilis* | -13.0 | Modern |
| Central American agouti | *Dasyprocta variegata* | -14.3 | Modern |
| Central American agouti | *Dasyprocta variegata* | -14.5 | Modern |
| Central American agouti | *Dasyprocta variegata* | -16.0 | Modern |
| Central American agouti | *Dasyprocta variegata* | -16.4 | Modern |
| Central American agouti | *Dasyprocta variegata* | -14.6 | Modern |
| Central American agouti | *Dasyprocta variegata* | -16.0 | Modern |
| Central American agouti | *Dasyprocta variegata* | -15.9 | Modern |
| Central American agouti | *Dasyprocta variegata* | -16.1 | Modern |
| Central American agouti | *Dasyprocta variegata* | -16.0 | Modern |
| Central American agouti | *Dasyprocta variegata* | -14.1 | Modern |
| Central American agouti | *Dasyprocta variegata* | -15.0 | Modern |
| Central American agouti | *Dasyprocta variegata* | -16.5 | Modern |
| Central American agouti | *Dasyprocta variegata* | -14.0 | Modern |
| pacarana | *Dinomys branickii* | -14.2 | Modern |
| pacarana | *Dinomys branickii* | -17.0 | Modern |
| pacarana | *Dinomys branickii* | -14.9 | Modern |
| capybara | *Hydrochoerus hydrochaeris* | 0.2 | Modern |
| capybara | *Hydrochoerus hydrochaeris* | -0.4 | Modern |
| capybara | *Hydrochoerus hydrochaeris* | 0.3 | Modern |
| capybara | *Hydrochoerus hydrochaeris* | -7.0 | Modern |
| capybara | *Hydrochoerus hydrochaeris* | -5.5 | Modern |
| capybara | *Hydrochoerus hydrochaeris* | -5.9 | Modern |
| Huallaga spiny rat | *Proechimys brevicauda* | -14.7 | Modern |
| Huallaga spiny rat | *Proechimys brevicauda* | -14.8 | Modern |
| Huallaga spiny rat | *Proechimys brevicauda* | -15.0 | Modern |
| Huallaga spiny rat | *Proechimys brevicauda* | -15.5 | Modern |
| Southern Amazon red squirrel | *Sciurus spadiceus* | -17.4 | Modern |
| Southern Amazon red squirrel | *Sciurus spadiceus* | -17.6 | Modern |
| Southern Amazon red squirrel | *Sciurus spadiceus* | -17.6 | Modern |
| Amazonian manatee | *Trichechus inunguis* | -11.5 | Modern |
| Amazonian manatee | *Trichechus inunguis* | -11.4 | Modern |
| Amazonian manatee | *Trichechus inunguis* | -13.6 | Modern |
| Amazonian manatee | *Trichechus inunguis* | -13.8 | Modern |
| Amazonian manatee | *Trichechus inunguis* | -13.2 | Modern |
| brown-throated thre-toed sloth | *Bradypus variegatus* | -16.1 | Modern |
| brown-throated thre-toed sloth | *Bradypus variegatus* | -15.8 | Modern |
| brown-throated thre-toed sloth | *Bradypus variegatus* | -15.9 | Modern |
| Linnaeus two-toed sloth | *Choloepus didactylus* | -17.0 | Modern |
| Linnaeus two-toed sloth | *Choloepus didactylus* | -17.2 | Modern |
| Hoffmanns two-toed sloth | *Choloepus hoffmanni* | -16.1 | Modern |
| Hoffmanns two-toed sloth | *Choloepus hoffmanni* | -16.4 | Modern |
| Hoffmanns two-toed sloth | *Choloepus hoffmanni* | -15.8 | Modern |
| Hoffmanns two-toed sloth | *Choloepus hoffmanni* | -15.5 | Modern |
| Hoffmanns two-toed sloth | *Choloepus hoffmanni* | -16.3 | Modern |
| Hoffmanns two-toed sloth | *Choloepus hoffmanni* | -15.6 | Modern |
| Hoffmanns two-toed sloth | *Choloepus hoffmanni* | -15.9 | Modern |
| Hoffmanns two-toed sloth | *Choloepus hoffmanni* | -15.2 | Modern |
| Hoffmanns two-toed sloth | *Choloepus hoffmanni* | -15.6 | Modern |
| Hoffmanns two-toed sloth | *Choloepus hoffmanni* | -15.1 | Modern |

## Supplementary Figures:

**Fig. A in S1:** Human bulk tooth δ^13^C_ap_ and δ^18^O for pre-colonial population from VGRX, by type of tooth analyzed


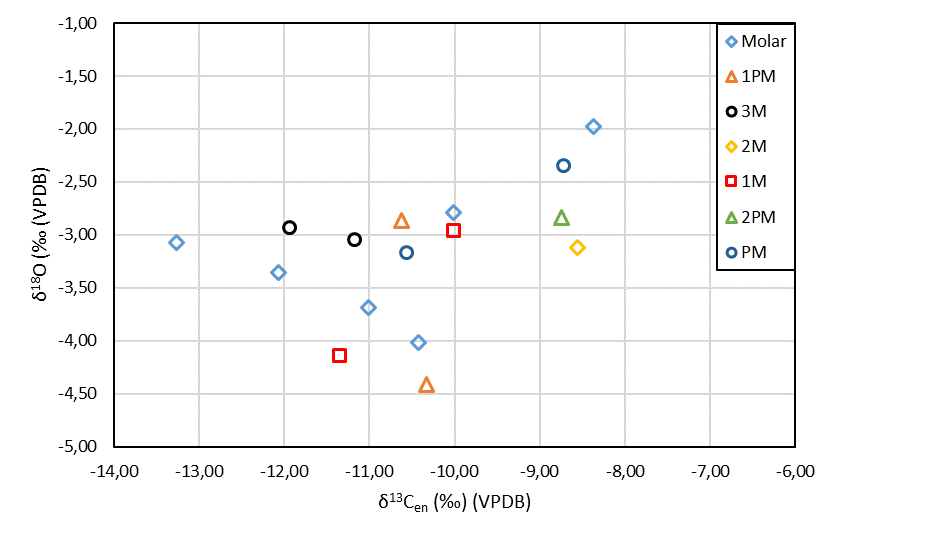


**Fig. B in S1:** Human bulk tooth δ^13^C_ap_ and δ^18^O for pre-colonial populations from VGRX, by sex.


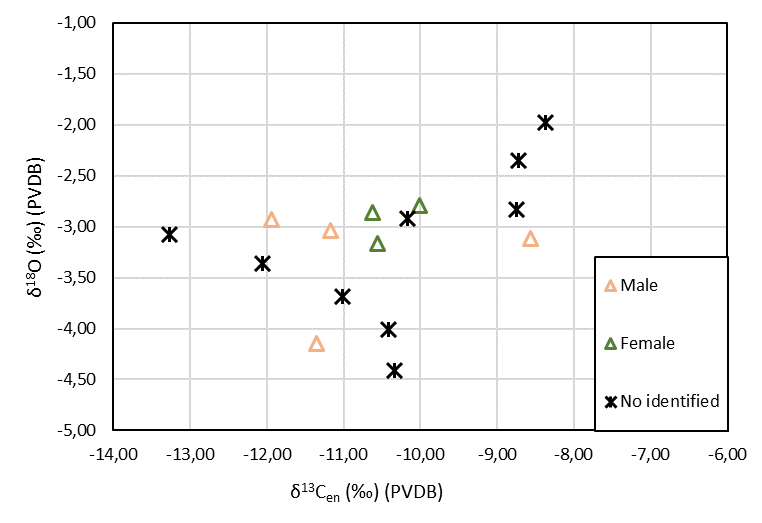


**Fig. C in S1:** Human bulk tooth δ^13^C_ap_ and δ^18^O for pre-colonial populations from VGRX, shown by type of burial


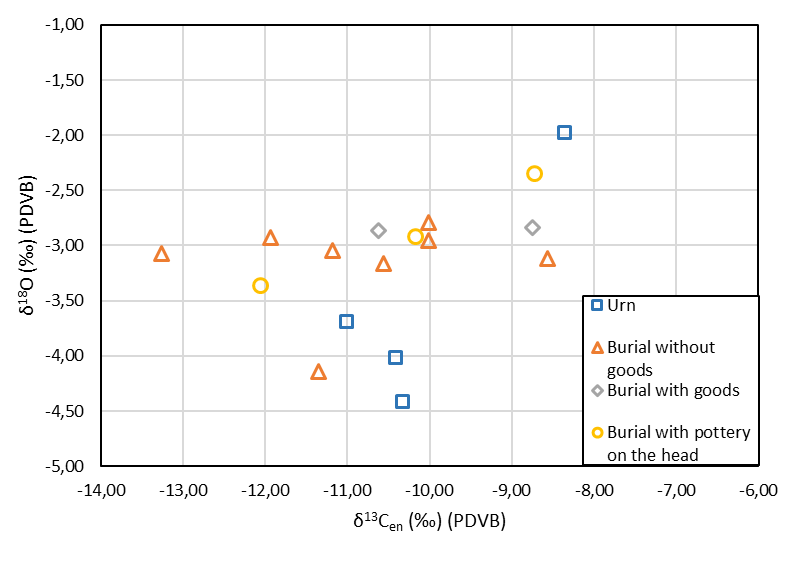


# References

x

| 1. | Lima HP, Fernandes GCB. Cerâmicas arqueológicas da Foz do Rio Xingu: uma primeira caracterização. In Barreto C, Lima HP, Betancourt CJ. Cerâmicas arqueológicas da Amazônia: Rumo a uma nova síntese. Belém: Iphan, Ministério da Cultura; 2016. p. 224-236. |
| --- | --- |
| 2. | Scientia Consultoria Científica. Arqueologia Preventiva nas Áreas de Intervenção da Usina Hidrelétrica de Belo Monte, Rio Xingu, PA. Relatório Parcial 11. São Paulo: Scientia Consultoria Científica; 2016b. |
| 3. | Scientia Consultoria Científica. Arqueologia Preventiva nas Áreas de Intervenção da Usina Hidrelétrica de Belo Monte, Rio Xingu, PA. Relatório Parcial 8. Sâo Paulo: Scientia Consultoria Científica; 2014b. |
| 4. | Castro AM. Um regime de opulência: grupos ceramistas na Volta Grande do Rio Xingu Belém: Universidade Federal do Pará; 2020. |
| 5. | Scientia Consultoria Científica. Arqueologia Preventiva nas Áreas de Intervenção da Usina Hidrelétrica de Belo Monte, Rio Xingu, PA. Relatório Parcial 7. São Paulo: Scientia Consultoria Científica; 2014a. |
| 6. | Scientia Consultoria Científica. Arqueologia Preventiva nas Áreas de Intervenção da Usina Hidrelétrica de Belo Monte, Rio Xingu, PA. Relatório Parcial 5. São Paulo: Scientia Consultoria Científica; 2013. |
| 7. | Castro AM, Müller LM, Heinen ILS, Kipnis R. The Koriabo pottery in the Volta Grande do Rio Xingu: the cases of the Sabiá 2 and Vila Rica 2 sites. In Barreto C, Lima H, Rostain S, Hofman C. Koriabo: From the Caribbean Sea to the Amazon River. Belém: Museu Paraense Emílio Goeldi; 2021. p. 203-225. |
| 8. | Scientia Consultoria Científica. Arqueologia Preventiva nas Áreas de Intervenção da Usina Hidrelétrica de Belo Monte, Rio Xingu, PA. Relatório Parcial 9. São Paulo: Scientia Consultoria Científica; 2015. |
| 9. | Scientia Consultoria Científica. Arqueologia Preventiva nas Áreas de Intervenção da Usina Hidrelétrica de Belo Monte, Rio Xingu, PA. Relatório Parcial 10. São Paulo: Scientia Consultoria Científica; 2016a. |
| 10. | Kipnis R, Caldarelli. SB. Caçadores-coletores do Holoceno Inicial no Médio Xingu. Especiaria- Caderno de Ciências Humanas. 2018. |
| 11. | Pereira ES. Levantamento arqueológico na área da UHE-Belo Monte, rio Xingu (PA). In ELETROBRÁS/ELETRONORTE , editor. CHE Belo Monte – Estudo de Impacto Ambiental. Meio Sócio-Econômico.; 2002. |
| 12. | Müller LM. Relatório de escavação da estrutura do sítio Bela Vista (PA-AL-26). Belém: MPEG; 2018. |
| 13. | Fernandes GCB, Lima HP, Ribeiro AB. Cerâmicas Koriabo e problematizações iniciais sobre a arqueologia na Foz do Rio Xingu.. Habitus. 2018: p. 403-424. |
| 14. | Robinson JG, Redford KH. Body Size, Diet, and Population Density of Neotropical Forest Mammals. The American Naturalist. 1986: p. 665–680. |
| 15. | Filho ARGF. Contribuição para uma estratégia de conservação de Alouatta ululata, nordeste do Brasil Lisboa: Universidade de Lisboa; 2016. |
| 16. | Botero-Arias R. Padrões de movimento, uso de microhabitat e dieta de jacaré-paguá, PaleosuchusPalpebrosus (crocodilia:alligatoridae), em uma floresta de paleovárzeaao sul do rio Solimões, Amazônia Central, Brazil Manaus: Instituto nacional de pesquisas da amazonia - INPA, Universidade Federal do Amazonas; 2007. |
| 17. | Jerozolimski A, Ribeiro MBN, Martins M. Are tortoises important seed dispersers in Amazonian forests? Oecologia. 2009: p. 517–528. |
| 18. | Yengle PJM. Effect of temperature and diet (starch and fiber) on embryonic development, energy metabolism and growth of red-footed tortoise (Chelonoidis carbonaria) Jaboticabal: Universidade Estadual Paulista "Júlio De Mesquita Filho" (UNESP) ; 2021. |
| 19. | Bizri HRE, Fa JE, Bowler M, Valsecchi J, Bodmer R, Mayor P. Breeding seasonality in the lowland paca (Cuniculus paca) in Amazonia: interactions with rainfall, fruiting, and sustainable hunting. Journal of Mammalogy. : p. 1101–1111. |
| 20. | Silvius KM, Fragoso JMV. Red-rumped Agouti (Dasyprocta leporina) Home Range Use in an Amazonian Forest: Implications for the Aggregated Distribution of Forest Trees. BIOTROPICA. : p. 74-83. |
| 21. | Jones KR, Lall KR, Garcia GW. Omnivorous Behaviour of the Agouti (Dasyprocta leporina): A Neotropical Rodent with the Potential for Domestication. Hindawi Scientifica. 2019; 2019: p. 1-5. |
| 22. | Dalponte JC, Tavares-Filho JA. Diet of the Yellow Armadillo, Euphractus sexcinctus, in South-Central Brazil. Edentata. 2004: p. 37-41. |
| 23. | Vaz VC, Santori RT, Jansen AM, Delciellos AC. Notes on Food Habits of Armadillos (Cingulata, Dasypodidae) and Anteaters. Edentata. : p. 84-89. |
| 24. | Tonetti AM, Biondi D. Dieta de capivara (Hydrochoerus hydrochaeris, Linnaeus, 1766) em ambiente urbano, Parque Municipal Tingui, Curitiba-PR. Acta Veterinaria Brasilica. 2015: p. 316-326. |
| 25. | Cardoso DC, deHart P, Freitas CEdC, Siqueira-Souza FK. Diet and ecomorphology of predator fish species of the Amazonian floodplain lakes. Biota Neotropica. 2019: p. 1-9. |
| 26. | Berndt A. Nutrição e ecologia nutricional de cervídeos brasileiros em cativeiro e no Parque Nacional das Emas - Goiás. Tese de doutorado ed. Piracicaba: Universidade de São Paulo-USP; 2005. |
| 27. | Mendes A. Fornecimento de uréia na dieta de catetos (Pecari tajacu) e uso de isótopo estável 15N como marcador para estimativa da síntese de nitrogênio microbiano Piracicaba: Universidade de São Paulo - USP; 2008. |
| 28. | Desbiez ALJ, Keuroghlian A, Beisiegel BdM, Medici EP, Gatti A, Pontes ARM, et al. Avaliação do Risco de Extinção do Cateto Pecari tajacu Linnaeus, 1758, no Brasil. Biodiversidade Brasileira. 2012: p. 74-83. |
| 29. | Tejada JV, Flynna JJ, Antoine PO, Pacheco V, Salas-Gismondi R, Cerling TE. Comparative isotope ecology of western Amazonian rainforest mammals. PNAS. 2020 October 20: p. 26263–26272. |
| 30. | Eisemberg CC, Reynolds SJ, Christian KA,VRC. Diet of Amazon river turtles (Podocnemididae): a review of the effects of body size, phylogeny, season and habitat. Zoology. : p. 92-100. |
| 31. | Wallace RB, Painter RLE. Observations on the Diet of the Giant Armadillo (Priodontes maximus Kerr, 1792). Edentata. 2013: p. 85-86. |
| 32. | Morais AA. Dieta Frugivora de Tapirus terrestris e deposição de fezes: contribuição para a dispersão de sementes e regeneração de florestas, Amazônia Central, AM Manaus: Universidade Federal do Amazonas; 2006. |
| 33. | Bradham J, Jorge MLSP, Pedrosa F, Keuroghlian A, Costa VE, Bercê W, et al. Spatial isotopic dietary plasticity of a Neotropical forest ungulate: the white-lipped peccary (Tayassu pecari). Journal of Mammalogy. 2019: p. 464–474. |
| 34. | Keuroghlian A, Desbiez ALJ, Beisiegel BdM, Medici EP, Gatti A, Pontes ARM, et al. Avaliação do Risco de Extinção do Queixada Tayassu pecari Link, 1795, no Brasil. Biodiversidade Brasileira. 2012: p. 3-11. |
| 35. | Bronk Ramsey C. Bayesian analysis of radiocarbon dates. Radiocarbon. 2009;(51): p. 337-360. |
| 36. | Reimer PJ, Bard E, Bayliss A, Beck JW, Blackwell PG, Ramsey CB, et al. INTCAL13 and MARINE13 Radiocarbon age calibration curves 0–50,000 years CAL BP. Radiocarbon. 2013: p. 1869–1887. |
| 37. | Hermenegildo T, Guapindaia VLC, Neves EG. New evidence for subsistence strategies of late pre-colonial societies of the mouth of the Amazon based on carbon and nitrogen isotopic data. Quaternary International 448. 2017 Agosto 20: p. 139-149. |
| 38. | van der Merwe NJ, Roosvelt AC, Vogel JC. Isotopic evidence for prehistoric subsistence change at Parmana, Venezuela. Nature. 1981: p. 536-538. |
| 39. | Roosevelt A. Resource Management in Amazonia before the Conquest: Beyond Ethnographic Projection. Advances in Economic Botany. 1989 June 27: p. 30-62. |
| 40. | Colonese AC, Winter R, Brandi R, Fossile T, Fernandes R, Soncin S, et al. Stable isotope evidence for dietary diversification in the pre-Columbian Amazon. Scientific Reports. 2020. |

x
